# Risk factors of under-five and infant mortality: An umbrella review of systematic reviews and meta-analyses

**DOI:** 10.7189/jogh.14.04260

**Published:** 2024-11-29

**Authors:** Bereket Kefale, Jonine Jancey, Amanuel T Gebremedhin, Sylvester Dodzi Nyadanu, Daniel G Belay, Gavin Pereira, Gizachew A Tessema

**Affiliations:** 1 Curtin School of Population Health, Curtin University, Perth, Western Australia, Australia; 2 Department of Reproductive Health, School of Public Health, Wollo University, Dessie, Ethiopia; 3 enAble Institute, Curtin University, Perth, Western Australia, Australia; 4 School of Nursing and Midwifery, Edith Cowan University, Perth, Western Australia, Australia; 5 School of Public Health, University of Adelaide, Adelaide, South Australia, Australia

## Abstract

**Background:**

Ensuring child survival is a critical global challenge, requiring a robust and comprehensive understanding of the risk factors contributing to under-five mortality (U5M). We aimed to synthesise and summarise the current available evidence on risk factors of U5M and infant mortality worldwide to inform global child health programmes.

**Methods:**

We searched six major databases (Embase, Medline, Scopus, CINAHL, Web of Science, and Global Health) and repositories of systematic reviews, as well as grey literature sources to identify systematic reviews and meta-analyses that examined the associations between risk factors of U5M and infant mortality between 1 January 1990 and 4 March 2024. The quality of reviews was assessed using A Measurement Tool to Assess Systematic Reviews, Version 2 (AMSTAR 2). The strength of evidence and direction of associations was graded.

**Results:**

Of 5684 records, we included 32 reviews (including five systematic reviews without meta-analysis) which comprised 1042 primary studies. We synthesised 28 and 29 unique risk factors associated with U5M and infant mortality, respectively. Although there was no convincing evidence for the risk factors, we found probable evidence of association between exclusive breastfeeding (consistent negative association), and maternal death (consistent positive association) with U5M. There was also probable evidence for the association of short (<18 months) interpregnancy intervals (less consistent negative association), pre-pregnancy maternal obesity (consistent positive association), and maternal HIV infection (consistent positive association) with infant mortality.

**Conclusions:**

While the review identified a broad range of risk factors, the overall evidence for most factors associated with under-five and infant mortality was ‘limited-suggestive’ or ‘limited and no conclusive’. Thus, further high-quality studies are required to strengthen the evidence on these risk factors.

**Registration:**

PROSPERO CRD42023455542.

Under-five mortality (U5M) is a critical marker of child survival and socioeconomic development [1,2]. Despite significant global progress, with a 58% reduction in infant mortality and a 62% reduction in U5M between 1990 and 2022, there are still nearly five million under-five children's deaths, which corresponds to an estimated 13 400 deaths every day [3]. These deaths primarily result from preventable and treatable causes, such as infectious diseases including pneumonia, diarrhoea, and malaria, as well as adverse birth outcomes. Nearly 75% of under-five deaths occur among infants [4,5].

In response to this ongoing challenge, various global and regional initiatives were developed, each with the overarching goal of reducing child mortality [6–12]. One such initiative is Sustainable Development Goal (SDG) 3, which specifically targets a reduction in U5M to ≤25 deaths per 1000 live births by 2030 [6]. However, early indications suggest that about 30% of countries worldwide are not likely to achieve these SDG targets by 2030 [13]. This underscores the urgent need for further action to accelerate progress in reducing U5M rates.

To address this critical issue and make advancements toward reducing U5M and infant mortality rates globally, it is crucial to have a comprehensive understanding of the risk factors contributing to these outcomes. Numerous studies have examined various factors associated with U5M [14–18] and infant mortality [14,17,19–21] and systematic reviews and meta-analyses have played a crucial role in objectively synthesising the scientific evidence [22–28]. However, these reviews exhibited variations in terms of quality, scope, and conclusions. While some reviews [22] examined broader risk factors such as sociodemographic and economic factors, maternal nutritional and reproductive factors, child-related factors, and environmental factors associated with U5M, the majority of systematic reviews and meta-analyses [24,29–34] focused on investigating the association of a single factor with U5M or infant mortality. However, a comprehensive umbrella review that synthesises and integrates these findings was lacking.

Therefore, this comprehensive umbrella review synthesised and summarised the current evidence on risk factors of U5M and infant mortality. The findings of this umbrella review may inform the design of effective strategies and guide further research that can significantly contribute to the global efforts to reduce U5M and infant mortality.

## METHODS

### Search strategy and selection criteria

We followed the preferred reporting items for systematic review and meta-analysis (PRISMA) 2020 reporting guideline (Table S1 in **Online Supplementary Document**) [35] and the Joanna Briggs Institute (JBI) Manual for evidence synthesis [36]. The review protocol was registered in the International Prospero Registry of Systematic Reviews and Meta-analysis (PROSPERO CRD42023455542) [37]. Articles were searched in six databases including Embase, Medline, Scopus, CINAHL, Web of Science, and Global Health. In addition, a complementary search was undertaken in systematic review repositories, such as the Cochrane Database of Systematic Reviews and Epistemonikos, as well as in Google Scholar for grey literature sources [38,39]. The reference lists of eligible reviews were also manually searched to identify potentially missed systematic reviews and meta-analyses from database searching. The search strategy consisted of both medical subject headings (MeSH) and keywords (Table S2 in the **Online Supplementary Document**). Searches were restricted to only English language reviews published since 1990. The search strategy was initially developed by BK, which was later reviewed and approved by the research team. The database search was initially undertaken on 6 November 2023, with an updated search being undertaken on 4 March 2024.

We included articles that met the following criteria:

1) population: under-five children and infants

2) exposure: reviews examined factors associated with all-cause U5M or infant mortality

3) outcome: U5M or infant mortality

4) study design: all systematic reviews, with or without meta-analyses, of quantitative studies.

Reviews were required to have clear research questions, conduct searches in at least one database, and include at least two studies for each factor outcome association. We included all reviews without any geographic restrictions. We did not include reviews on cause-specific U5M or infant mortality, or those focusing on special populations. Reviews on immunisation, supplementation, nutritional interventions, and other public health interventions were excluded. We also excluded reviews based primarily on theoretical studies or opinions, as well as narrative reviews, mapping reviews, scoping reviews, and rapid reviews. Records retrieved from electronic databases were exported to EndNote 20, duplicates were removed, and the remaining records were imported into Rayyan for screening [40]. Two reviewers (BK and DGB) independently assessed the eligibility, with discrepancies resolved by a third reviewer (GAT).

### Data extraction and quality appraisal

Two investigators (BK and DGB) extracted data using a standardised data extraction form adapted from the JBI data extraction form for reviews [36]. For each review, we extracted: the first author name, publication date ranges of primary studies, database searching dates, region, number of primary studies, number of databases searched, use of grey literature, protocol registration status, review guideline used, review type, heterogeneity level, publication bias assessment, outcomes measured, and reported association magnitudes (relative risk (RR), odds ratio (OR), hazard ratio (HR), and β coefficient with 95% confidence intervals (CIs)). The quality of reviews was evaluated using The quality of reviews was assessed using A Measurement Tool to Assess Systematic Reviews, Version 2 (AMSTAR 2) [41], and two reviewers (BK and DGB) conducted the appraisal. Any disagreements among reviewers were resolved through discussion.

### Data analysis

The extracted data from the included reviews were summarised using narrative synthesis and summary tables. The inclusion of studies in multiple reviews was assessed using citation matrices with the corrected covered area (CCA) algorithm [42]. The direction of the association and the strength of evidence from the systematic reviews with meta-analysis were assessed using grading approaches, following the approaches of previously published umbrella review [43–45]. The direction of each association was categorised into consistent positive (++), less consistent positive (+), consistent negative (−−), less consistent negative (−), unclear or contradictory (?), less consistent null (0) and consistent null association (00), based on the consistency of the association within reviews, or the included primary articles (Table S3 in the **Online Supplementary Document**) [43]. The confidence in the evidence was rated and summarised by considering the consistency and strength of association, precision, heterogeneity, and the number and quality of pooled primary studies. It was classified as convincing evidence, probable evidence, limited-suggestive evidence, and limited, no conclusive evidence (Table S4 in the **Online Supplementary Document****)** [44,45]. We used RR by converting or assuming the OR approximates RR [46] (Table S5 in the **Online Supplementary Document**). Relative risk values were also standardised for all air pollutants to represent a 10 µg per cubic metre (μg/m^3^) increase, except for carbon monoxide, which was measured per one milligram per cubic metre (mg/m^3^) increase [47,48] (Table S6 in the **Online Supplementary Document**). We used forest plots to intuitively present the effect measures identified in the included systematic reviews and meta-analyses.

## RESULTS

**Figure 1:**
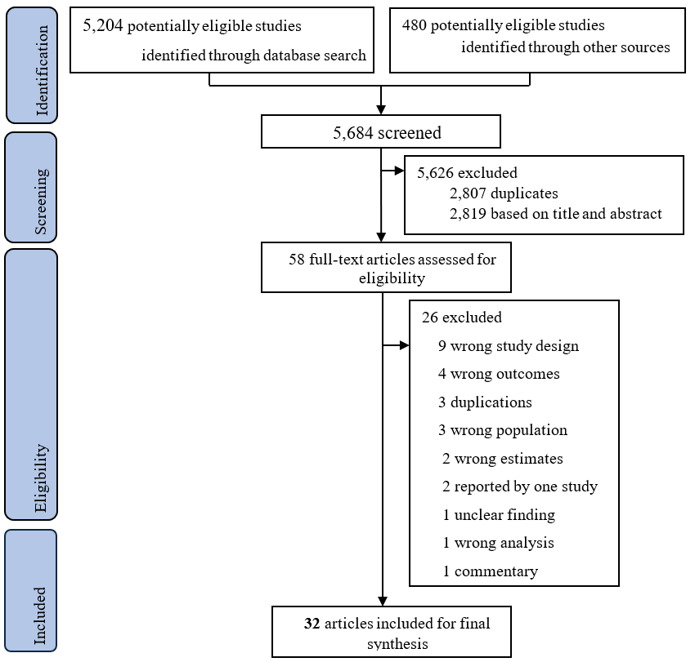
Study selection.

We identified a total of 5684 records from major databases, systematic review repositories, and grey literature sources. Of these, 2807 duplicate records were removed, and a further 2819 records were excluded based on abstract and title screening. After a full-text review of 58 articles, 32 eligible systematic reviews and meta-analyses were included [22–34, 49–67] (n = 5 were systematic reviews only) [22,24,26,30,55]. Twenty-six articles were excluded with the reasons detailed in Table S7 in the **Online Supplementary Document**. Of the included reviews, 10 reviews [22–26,33,49–52] focused on U5M, and the remaining 24 reviews [23,26–32,34,53–67] addressed infant mortality (**Figure 1**).

The 32 reviews [22–34,49–67] were published from August 1998 [54] to January 2024 [26], with more than two-thirds (21 reviews) [22–24,26–30,32,33,49–53,56,57,60,61,63,66] published after 2015. These reviews collectively examined a total of 1042 primary studies, which were published over the past five decades (1972–2022). Among these, 719 primary studies, including more than 67 million participants, focused on U5M or infant mortality. While 18 reviews (56%) were conducted at the global level [22,24,25,29,31,33,49,53–57,60–62,64,65], and six (19%) reviews were conducted at national level. Nineteen reviews [22–24,26–30,32–34,49,51,52,57,61–64], followed the PRISMA guidelines for reporting. Moreover, 29 reviews examined the association of a single risk factor domain [23–25,28–34,49–67], and under-five and/or infant mortality (**Table 1**, Table S8 in the **Online Supplementary Document**).

**Table 1:** Summary characteristics of included systematic reviews and meta-analyses (n = 32)

Characteristics		Studies, n	Systematic reviews and meta-analyses
**Outcomes studied**	U5M	8	Bhusal 2022 [22], Balaj 2021 [24], Garoma 2011 [25], Chikhungu 2017 [33], Karimi 2020 [49], Forde 2017 [50], Huang 2017 [51], Pretorius 2020 [52]
	Infant mortality	22	Eltayib 2023 [27], Karami 2024 [26], Kiross 2019 [28], Adane 2021 [29], Ahrens 2018 [30], Bagade 2018 [32], Aune 2014 [31], Dadi 2015 [34], Brennan 2016 [53], Brocklehurst 1998 [54], Glinianaia 2004 [55], Huo 2021 [56], Jacques 2019 [57], Jahan 2007 [58], Kozuki 2013 [59], Luben 2023 [60], Mazzone 2023 [61], Meehan 2014 [62], Nguyen 2019 [63], Quansah 2015 [64], Sankar 2015 [65], Rahman 2021 [66], Weightman 2012 [67]
	Both	2	Islam 2022 [23], Karami 2024 [26]
**Year of publication**	<2000	1	Brocklehurst 1998 [54]
	2000–2015	10	Glinianaia 2004 [55], Jahan 2007 [58], Garoma 2011 [25], Weightman 2012 [67], Kozuki 2013 [59], Aune 2014 [31], Meehan 2014 [62], Dadi 2015 [34], Quansah 2015 [64], Sankar 2015 [65]
	>2015	21	Brennan 2016 [53], Forde 2017 [50], Huang 2017 [51], Chikhungu 2017 [33], Ahrens 2018 [30], Bagade 2018 [32], Jacques 2019 [57], Kiross 2019 [28], Nguyen 2019 [63], Karimi 2020 [49], Pretorius 2020 [52], Adane 2021 [29], Balaj 2021 [24], Huo 2021 [56], Rahman 2021 [66], Bhusal 2022 [22], Islam 2022 [23], Eltayib 2023 [27], Luben 2023 [60], Mazzone 2023 [61], Karami 2024 [26]
**Geographic focus of reviews**	Global focus	18	Balaj 2021 [24], Bhusal 2022 [22], Chikhungu 2017 [33], Garoma 2011 [25], Karimi 2020 [49], Adane 2021 [29], Aune 2014 [31], Bagade 2018 [32], Brennan 2016 [53], Brocklehurs 1998 [54], Glinianaia 2004 [55], Huo 2021 [56], Jacques 2019 [57], Luben 2023 [60], Mazzone 2023 [61], Meehan 2014 [62], Quansah 2015 [64], Sankar 2015 [65], Nguyen 2019 [63], Quansah 2015 [64], Sankar 2015 [65]
	Regional focus	8	Forde 2017 [50], Pretorius 2020 [52], Islam 2022 [23], Ahrens 2018 [30], Eltayib 2023 [27], Jahan 2007 [58], Kozuki 2013 [59], Nguyen 2019 [63],
	National focus	6	Huang 2017 [51], Karami 2024 [26], Dadi 2015 [34], Kiross 2019 [28], Rahman 2021 [66], Weightman 2012 [67]
**Number of primary studies included**	<10	17	Chikhungu 2017 [33], Huang 2017 [51], Pretorius 2020 [52], Ahrens 2018 [30], Aune 2014 [31], Bagade 2018 [32], Brocklehurs 1998 [54], Dadi 2015 [34], Glinianaia 2004 [55], Jacques 2019 [57], Jahan 2007 [58], Kiross 2019 [28], Kozuki 2013 [59], Nguyen 2019 [63], Quansah 2015 [64], Sankar 2015 [65], Weightman 2012 [67]
	10–20	3	Adane 2021 [29], Garoma 2011 [25], Mazzone 2023 [61],
	20–30	9	Bhusal 2022 [22], Forde 2017 [50], Karimi 2020 [49], Islam 2022 [23], Brennan 2016 [53], Eltayib 2023 [27], Huo 2021 [56], Luben 2023 [60], Meehan 2014 [62]
	>30	3	Balaj 2021 [24], Karami 2024 [26], Rahman 2021 [66]
**Language applied for literature search in the included reviews**	Only English	15	Adane 2021 [29], Ahrens 2018 [30], Bagade 2018 [32], Brennan 2016 [53], Bhusal 2022 [22], Dadi 2015 [34], Chikhungu 2017 [33], Garoma 2011 [25], Glinianaia 2004 [55], Islam 2022 [23], Jahan 2007 [58], Luben 2023 [60], Meehan 2014 [62], Nguyen 2019 [63], Sankar 2015 [65],
	Any language	6	Balaj 2021 [24], Huo 2021 [56], Karimi 2020 [49], Mazzone 2023 [61], Pretorius 2020 [52], Weightman 2012 [67]
	English plus one or more other languages	5	Eltayib 2023 [27], Forde 2017 [50], Huang 2017 [51], Jacques 2019 [57], Karami 2024 [26]
	Not reported	6	Aune 2014 [31], Brocklehurs 1998 [54], Glinianaia 2004 [55], Kiross 2019 [28], Kozuki 2013 [59], Quansah 2015 [64], Rahman 2021 [66]
**Use of PRISMA guidelines**	Yes	19	Adane 2021 [29], Ahrens 2018 [30], Bagade 2018 [32], Balaj 2021 [24], Bhusal 2022 [22], Chikhungu 2017 [33], Dadi 2015 [34], Eltayib 2023 [27], Huang 2017 [51], Islam 2022 [23], Jacques 2019 [57], Karami 2024 [26], Karimi 2020 [49], Kiross 2019 [28], Mazzone 2023 [61], Meehan 2014 [62], Nguyen 2019 [63], Pretorius 2020 [52], Quansah 2015 [64]
	No	13	Aune 2014 [31], Brennan 2016 [53], Brocklehurs 1998 [54], Forde 2017 [50], Garoma 2011 [25], Glinianaia 2004 [55], Huo 2021 [56], Jahan 2007 [58], Kozuki 2013 [59], Luben 2023 [60], Rahman 2021 [66], Sankar 2015 [65], Weightman 2012 [67]
**Evidence of pre-specified protocol registration**	Yes	8	Adane 2021 [29], Ahrens 2018 [30], Bagade 2018 [32], Balaj 2021 [24], Brocklehurs 1998 [54], Karimi 2020 [49], Mazzone 2023 [61], Meehan 2014 [62]
	No	24	Aune 2014 [31], Bhusal 2022 [22], Brennan 2016 [53], Chikhungu 2017 [33], Dadi 2015 [34], Eltayib 2023 [27], Forde 2017 [50], Garoma 2011 [25], Glinianaia 2004 [55], Huang 2017 [51], Huo 2021 [56], Islam 2022 [23], Jacques 2019 [57], Jahan 2007 [58],Karami 2024 [26], Kiross 2019 [28] Kozuki 2013 [59], Luben 2023 [60], Nguyen 2019 [63], Pretorius 2020 [52], Quansah 2015 [64], Rahman 2021 [66], Sankar 2015 [65], Weightman 2012 [67]

U5M – under-five mortality

### Quality appraisal

Of the total reviews, only five [24,29,30,49,62] were rated as moderate quality, as they did not have critical weaknesses. Eighteen reviews [23,26–28,31–33,51–54,56,57,60,61,63,64,67] had low quality, while nine reviews [22,25,34,50,55,58,59,65,66] were critically low in quality. The most common critical weakness identified among the reviews was the absence of protocol registration (n = 24) [22,23,25–28,31–34,50–53,55–60,63,64,66,67] followed by the failure to assess publication bias (n = 5) [54,58,59,61,65] (Figure S1 in the **Online Supplementary Document**).

### Risk factors of under-five mortality

**Figure 2:**
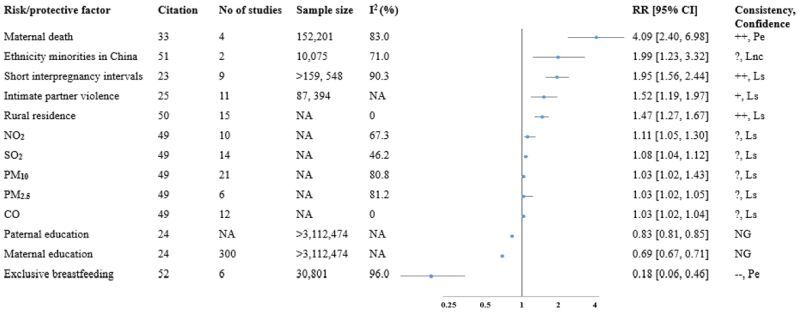
Forest plot for summary of evidence of meta-analyses on U5M. ‘++’ indicates consistent positive association, ‘+’ indicates less consistent positive association, ‘?’ indicates unclear or contradictory direction. CO – carbon monoxide, Lnc – limited no conclusive evidence, Ls – limited-suggestive evidence, NA – not available, NG – not graded, NO_2_ – nitrogen dioxide, Pe – probable evidence, PM_2.5_ – particulate matters with aerodynamic diameter ≤2.5μm, PM_10_ – particulate matters with aerodynamic diameter ≤10μm, SO_2_ – sulphur dioxide.

Ten reviews [22–26,33,49–52] (two systematic reviews only) [22,26] reported a total of 28 unique factors associated with U5M. Among these factors, 21 were exclusively reported in individual reviews. These factors were classified as sociodemographic and economic (n = 11), maternal reproductive health-related and maternal death (n = 10), child-related (n = 4), and environmental factors (n = 3). The most frequently investigated factor was maternal education, which was reported in three reviews (**Table 2**, Table S9 in the **Online Supplementary Document**) [22,24,26]. We identified nine factors, and 20 associations across eight meta-analyses [23–25,33,49–52], with each association being reported by a single meta-analysis. However, we could only grade the evidence of 12 associations, as eight lacked key details to allow grading for the observed associations. We found probable evidence for the association of exclusive breastfeeding (EBF) and maternal death with U5M. Other observed associations were rated as limited-suggestive or limited or no conclusive evidence. We found consistent positive associations for rural residence and maternal death (**Figure 2**, Table S10 in the **Online Supplementary Document**).

**Table 2:** Factors associated with U5M

Category	Risk/protective factor	Citation(s)	Primary studies, n
**Sociodemographic and economic factors**	Age at birth of the child	Bhusal 2022 [22], Karami 2024[26]	12
	Maternal education	Bhusal 2022 [22], Karami 2024 [26], Balaj 2021 [24]	317
	Paternal education	Karami 2024 [26], Balaj 2021 [24]	NA
	Maternal employment status	Bhusal 2022 [22]	5
	Poor wealth status	Bhusal 2022 [22]	4
	Marital status (single)	Bhusal 2022 [22]	3
	Rural residence	Bhusal 2022 [22], Forde 2017 [50], Karami 2024 [26]	37
	Ethnicity	Bhusal 2022 [22], Huang 2017 [51]	2
	Religion (Muslim)	Bhusal 2022 [22]	3
	Family size	Bhusal 2022 [22]	4
	Region	Bhusal 2022 [22]	5
**Reproductive health-related and maternal death**	Age at first birth (<18 y)	Bhusal 2022 [22]	3
	Intimate partner violence	Garoma 2011 [25]	11
	Antenatal care utilisation	Bhusal 2022 [22]	4
	Contraceptive use	Bhusal 2022 [22]	4
	Short interpregnancy intervals (<18 or 24 mo)	Bhusal 2022[22], Islam 2022 [23]	15
	Multiple pregnancies	Bhusal 2022 [22]	7
	Caesarean section delivery	Bhusal 2022 [22]	3
	Home delivery	Bhusal 2022 [22]	2
	History of child loss	Bhusal 2022 [22]	2
	Maternal death	Chikhungu 2017 [33]	4
**Child-related factors**	Sex of a child (female)	Bhusal 2022 [22]	7
	Breastfeeding status (breastfed)^a^	Bhusal 2022 [22], Pretorius 2020 [52]	
	Birth order	Bhusal 2022 [22]	6
	Low birth weight	Bhusal 2022 [22]	6
**Environmental factors**	Type of toilet (no/poor)	Bhusal 2022 [22]	2
	Source of energy for cooking	Bhusal 2022 [22]	4
	Air pollutant		
	*PM_10_*	Karimi 2020 [49]	6
	*PM_2.5_*	Karimi 2020 [49]	21
	*CO*	Karimi 2020 [49]	12
	*SO_2_*	Karimi 2020 [49]	14
	*NO_2_*	Karimi 2020 [49]	10

CO – carbon monoxide, NO_2_ – nitrogen dioxide, PM_2.5_ – particulate matters with aerodynamic diameter ≤2.5μm, PM_10_ – particulate matters with aerodynamic diameter ≤10μm, SO_2_ – sulphur dioxide

#### Sociodemographic factors

Three meta-analyses [24,50,51] reported four sociodemographic factors such as maternal education, paternal education, residence, and ethnicity. A meta-analysis [24] showed a 31% reduction in U5M (RR = 0.69; 95% CI = 0.67, 0.71) among children of mothers who completed secondary education compared to those of non-educated mothers. Similarly, children of fathers who completed secondary school had a 17% decreased risk of U5M (RR = 0.83; 95% CI = 0.81, 0.85) compared with those children born to non-educated fathers.

Residence was examined in a meta-analysis [50] of 15 studies, which showed a 47% higher risk of U5M (RR = 1.47; 95% CI = 1.27, 1.67) among children residing in rural areas. The evidence was graded as limited-suggestive, with a consistent positive association. Another meta-analysis [51] involving two studies (n = 10 075 participants) found two times increased risk of U5M among children of women from ethnic minority groups compared to the Han ethnic community in China (RR = 2.02; 95% CI = 1.23, 3.32). However, the evidence showed unclear or contradictory directions, with limited and no conclusive evidence (**Figure 2**, Table S10 in the **Online Supplementary Document**).

#### Maternal and child-related factors

Four meta-analyses [23,25,33,52] examine four factors associated with U5M including maternal death, intimate partner violence against women (IPV), short (<24 months) interpregnancy intervals (IPIs), and EBF. We found probable evidence for maternal death and EBF, while the evidence for IPV and short IPV was limited-suggestive. We observed a consistent positive association between short (<24 months) IPIs and maternal death with U5M. Maternal death was examined in a meta-analysis [33] of four cohort studies (n = 152 201 participants) and found a 4-fold increased risk of U5M associated with maternal death (RR = 4.09; 95% CI = 2.40, 6.98), with a consistent positive association and probable evidence. A meta-analysis [52] of six studies (n = 30 801 participants), revealed an 82% reduction in U5M among exclusively breastfed infants (RR = 0.18; 95% CI = 0.06, 0.46). The evidence showed a consistent negative association and was graded as probable evidence. A meta-analysis [23] of nine studies (n >159 548 participants) found that children with short (<24 months) IPIs had almost a two times increased risk of U5M (RR = 1.95; 95% CI = 1.56, 2.44) compared to children with long (≥24 months) IPIs. The evidence showed a consistent positive association and was graded as limited-suggestive. Lifetime exposure to IPV [25] and U5M was examined in 11 studies (n = 87 394 participants). The finding showed a 52% increased risk of U5M among children of mothers who experienced IPV (RR = 1.52; 95% CI = 1.19, 1.96). The overall direction was a less consistent positive association with limited suggestive (**Figure 2**, Table S10 in the **Online Supplementary Document**).

#### Environmental factors

The risk of U5M associated with exposure to ambient air pollutants such as particulate matter (PM_2.5_ and PM_10_), carbon monoxide (CO), sulphur dioxide (SO_2_), nitrogen dioxide (NO_2_), and ozone (O_3_) were reported in a meta-analysis [49] based on 6–21 primary studies. However, the overall findings revealed unclear and contradictory direction of associations with limited-suggestive evidence (**Figure 2**, Table S10 in the **Online Supplementary Document**).

### Risk factors of infant mortality

A total of 24 reviews [23,26–32,34,53–67], (four systematic reviews only) [26,30,32,55] reported 29 factors associated with infant mortality. These factors were categorised as sociodemographic and economic (n = 6), maternal reproductive health-related and nutrition (n = 9), maternal morbidity and mortality (n = 4), child-related (n = 8), and environmental factors (n = 2). Three factors such as IPV, history of stillbirth, and history of abortion were reported in systematic reviews that did not include meta-analyses. The most frequently reported factor was short IPIs (n = 4) [23,30,34,59] followed by maternal education (n = 3) [26–28] and socioeconomic status (n = 3) [26,58,67] (**Table 3**, Table S9 in the **Online Supplementary Document**).

**Table 3:** Summary of factors associated with infant mortality

Category	Risk/protective factor	Citation(s)	Primary studies, n
**Sociodemographic and economic factors**	Maternal education	Eltayib 2023 [27], Kiross 2019 [28], Karami 2024 [26]	14
	Paternal education	Eltayib 2023 [27], Karami 2024 [26]	7
	Paternal unemployment	Eltayib 2023 [27]	2
	Consanguineous marriage	Eltayib 2023 [27]	2
	Rural residence	Rahman 2021 [66], Karami 2024 [26]	68
	Poor socioeconomic status	Jahan 2007 [58], Weightman 2012, Karami 2024 [26]	21
**Reproductive health and nutrition**	Short interpregnancy interval (<24 or <18 mo)	Dadi 2015 [34], Kozuki 2013 [59], Islam 2022 [23], Ahrens 2018 [30]	25
	Intimate partner violence	Bagade 2018 [32]	2
	Multiple pregnancies	Eltayib 2023 [27]	2
	Vaginal delivery	Eltayib 2023 [27]	2
	Antenatal care utilisation	Eltayib 2023 [27]	2
	History of stillbirth	Karami 2024 [26]	3
	History of abortion	Karami 2024 [26]	2
	Maternal overweight	Huo 2021 [56]	8
	Maternal obesity	Huo 2021 [56], Meehan 2014 [62]	18
**Maternal morbidity and mortality**	Maternal mental illness		
	*Maternal anxiety and depression*	Adane 2021 [29]	5
	*Severe mental illness*	Adane 2021 [29]	7
	*Postnatal depression*	Jacques 2019 [57]	3
	Maternal epilepsy	Brocklehurst 1998 [54]	13
	Maternal HIV infection	Mazzone 2023 [61]	9
	Maternal death	Nguyen 2019 [63]	2
**Child-related factors**	Female infant	Eltayib 2023 [27]	4
	Preterm birth (<37 weeks)	Eltayib 2023 [27]	6
	Low birthweight (<2500 g)	Eltayib 2023 [27]	3
	Small for gestational age	Eltayib 2023 [27]	2
	Low Apgar score (1 and/or 5 min)	Eltayib 2023 [27]	2
	Breech presentation	Eltayib 2023 [27]	2
	Exclusive breastfeeding	Sankar 2015 [65]	2
	Infant exposure to HIV	Brennan 2016 [53]	22
**Environmental factors**	Arsenic exposure	Quansah 2015 [64]	5
	Air pollutant		
	*SO_2_ (1 ppm)*	Luben 2023 [60]	8
	*NO_2_ (1 ppm)*	Luben 2023 [60]	11

NO_2_ – nitrogen dioxide, SO_2_ – sulphur dioxide

**Figure 3:**
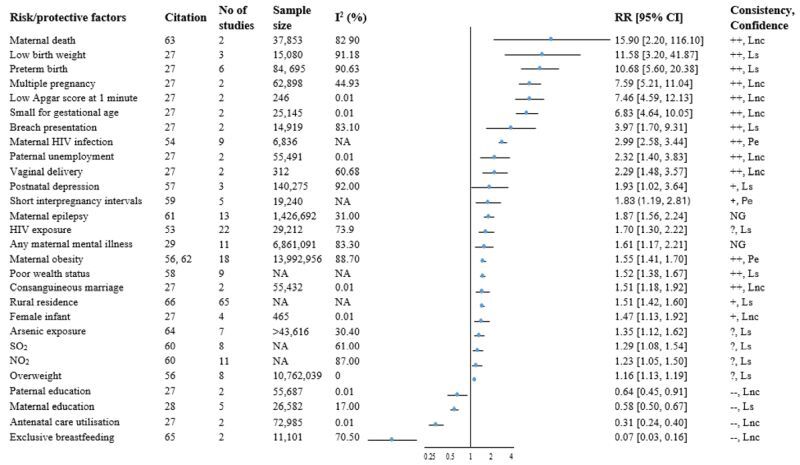
Forest plot for summary of evidence of meta-analyses on infant mortality. ‘++’ indicates consistent positive association, ‘+’ indicates less consistent positive association, ‘?’ indicates unclear or contradictory direction; ‘–’ indicates consistent negative association. For factors reported by two meta-analyses, the higher estimate was reported. CO – carbon monoxide, Lnc – limited no conclusive evidence, Ls – limited-suggestive evidence, NA – not available, NG – not graded, NO_2_ – nitrogen dioxide, O_3_ – ozone; Pe - probable evidence.

This umbrella review synthesised a total of 26 factors and 52 associations from 20 meta-analyses [23,27–29,31,34,53,54,56–67]. Of these, only two associations were examined in two meta-analyses, while the rest were reported in a single meta-analysis each. We found an increased risk of infant mortality in 36 associations. A total of 46 associations were graded, while six lacked essential details. Among the graded associations, five had probable evidence, 26 had limited-suggestive evidence, and 15 had limited and no conclusive evidence. Eighteen associations showed consistent positive associations (**Figure 3**, Table S11 in the **Online Supplementary Document**).

#### Sociodemographic and economic factors

Five meta-analyses [27,28,58,66,67] reported factors, including maternal education, paternal education, maternal employment, paternal employment, consanguineous marriage, rural residence, and socioeconomic status. The evidence for all factors was ‘limited-suggestive’ or ‘limited and no conclusive’. Maternal education and paternal education showed consistent negative associations with infant mortality, while we observed consistent positive associations for paternal unemployment and consanguineous marriage. A meta-analysis of two studies (n = 26 582 participants) showed a 42% lower risk of infant mortality among mothers with secondary education or above compared to non-educated mothers (RR = 0.58; 95% CI = 0.50, 0.67). The evidence was graded as limited suggestive with a consistent negative association (**Figure 3**, Table S5 in the **Online Supplementary Document**). Paternal education was reported in another meta-analysis [27], using two studies (n = 55 687 participants), which showed a 36% decreased risk of infant mortality among children of secondary or higher educated fathers compared to children of fathers with primary or lower education (RR = 0.64; 95% CI = 0.45, 0.91). While the observed association was consistent negative, the evidence was limited and no conclusive. Paternal employment was examined in a meta-analysis [27] of two studies (n = 55 491 participants) and revealed a 2.3 times increased risk of infant mortality among infants of unemployed fathers (RR = 2.32; 95% CI = 1.40, 3.83). It showed a consistent positive association but with limited and no conclusive evidence.

A meta-analysis [27] consisting of two studies (n = 55 432 participants), examined the risk of infant mortality in consanguineous marriage. It revealed a 51% increased risk of infant mortality with a consistent positive association, although the evidence is limited and no conclusive (RR = 1.51; 95% CI = 1.18, 1.92). Two meta-analyses [58,67] found an increased risk of infant mortality in families with poor socioeconomic status with limited-suggestive evidence (**Figure 3**, Table S11 in the **Online Supplementary Document**).

#### Maternal nutrition and reproductive health-related factors

A total of six factors were examined in seven meta-analyses [23,27,31,34,56,59,62]. These included IPIs, multiple pregnancies, gravidity, vaginal delivery, antenatal care utilisation, and maternal nutritional status. We found probable evidence for short (<18 months) IPIs and maternal pre-pregnancy obesity. IPI was examined in three meta-analyses [23,34,59]. A meta-analysis [59] of five cohort studies showed an 83% increased risk of infant mortality among IPIs of less than 18 months (RR = 1.83; 95% CI = 1.19, 2.81). The overall evidence was rated as probable evidence with a less consistent positive association. Two meta-analyses [23,34] conducted in low- and middle-income countries (LMICs) also found approximately two times increased risk of infant mortality in short (<24 months) IPIs, with a consistent positive association (RR = 1.92; 95% CI = 1.77, 2.07). However, the evidence was rated as limited-suggestive. A meta-analysis [27] found an increased risk of infant mortality among vaginal deliveries and multiple pregnancies with a consistent positive association, although the evidence was limited and no conclusive. Mothers who used antenatal care had a 69% decreased risk of infant mortality (RR = 0.31; 95% CI = 0.24, 0.40), with a consistent negative association but limited and no conclusive evidence [27].

The association between maternal nutrition status and risk of infant mortality was reported in three meta-analyses [31,56,62]. A meta-analysis [56] of eight cohort studies (n = 10 762 039 participants) investigated the risk of infant mortality among overweight (body mass index (BMI)≥25 kg/m^2^) women and showed a 16% increased risk of infant mortality compared to healthy weight women (RR = 1.16; 95% CI = 1.13, 1.19). However, the evidence showed unclear or contradictory direction and was limited-suggestive evidence. Two meta-analyses [56,62] including 18 studies (n = 139 922 956 participants) reported the association between maternal pre-pregnancy obesity (BMI≥30 kg/m^2^) and infant mortality, and the largest RR was 1.55 (95% CI = 1.41, 1.70). We found a consistent positive association with probable evidence. A meta-analysis [62] of three studies (n = 1 423 013 participants) also found a 2-fold increased risk of infant mortality among class II (BMI≥35kg/m^2^) obese women. However, the association of underweight and overweight with infant mortality showed unclear or contradictory direction (**Figure 3**, Table S11 in the **Online Supplementary Document**).

#### Maternal morbidity and mortality

The associations between maternal mental illness, epilepsy, maternal HIV, and maternal death with infant death were reported in five meta-analyses [29,54,57,61,63]. We found probable evidence for only maternal HIV infection. We observed consistent positive associations for maternal death and maternal HIV infection. A meta-analysis [29] of 11 studies (n = 6 861 091 participants) found a 61% increased risk of infant mortality among mothers with any mental illness (RR = 1.61; 95% CI = 1.17, 2.21, *I*^2^ = 83.3%). Moreover, mothers with postnatal depressive symptoms or depression had about 2-fold increased risk of infant mortality (RR = 1.93; 95% CI = 1.02, 1.64) [57]. It showed a less consistent positive association with no conclusive evidence. The risk of infant mortality among epileptic mothers was examined in a meta-analysis [61] of 13 studies (n = 1 426 692 participants) and showed an 87% increased risk of infant mortality (RR = 1.87; 95% CI = 1.56, 2.24, *I*^2^ = 31%).

A meta-analysis [54] of nine cohort studies (n = 6836 participants) found about three times higher risk of infant mortality among HIV-infected mothers (RR = 2.99; 95% CI = 2.58, 3.44). It showed a consistent positive association, with probable evidence. The association of maternal death and infant mortality was examined in a meta-analysis [63] of two studies that showed a consistent positive association, though, the evidence was limited and no conclusive (**Figure 3**, Table S11 in the **Online Supplementary Document**).

#### Infant-related factors

Three meta-analyses [27,53,65] examined eight infant-related factors such as infant sex, preterm birth, low birth weight, small for gestational age, Apgar score, breach presentation, breastfeeding status, and exposure to HIV. The evidence for all factors was limited-suggestive or limited and no conclusive. Most factors showed consistent positive associations, while EBF showed a consistent negative association.

A meta-analysis [27] involving four studies (n = 465 participants) revealed a 47% increased risk of infant mortality among female infants (RR = 1.47; 95% CI = 1.13, 1.92, *I*^2^ = 0.01%), showed a less consistent positive association. Preterm birth was examined in a meta-analysis [27] of six studies (n = 84 695 participants) and showed a consistent positive association. We also found an increased risk of infant mortality with a consistent positive association among infants with low birth weight, small for gestational age, and low Apgar score.

The risk of infant mortality among HIV-exposed uninfected infants was examined in a meta-analysis [53] of 22 cohort studies (n = 29 212 participants). HIV-exposed uninfected infants had a 70% increased risk of mortality, compared to unexposed infants (RR = 1.70; 95% CI = 1.30, 2.22). However, the association showed unclear or contradictory directions with limited suggestive evidence. A meta-analysis [65] of two studies (n = 11 101 participants) showed a 93% decreased risk of infant mortality among exclusively breastfed infants, indicating a consistent negative association, although the evidence was limited and no conclusive (RR = 0.07; 95% CI = 0.03, 0.16) (**Figure 3**, Table S11 in the **Online Supplementary Document**).

#### Environmental factors

Two meta-analyses [60, 64] identified the association of environmental factors with infant mortality. A meta-analysis [64] found a 35% increased risk of infant mortality associated with arsenic exposure (RR = 1.35; 95% CI = 1.12, 1.62). The evidence showed unclear or contradictory direction with limited suggestive evidence. Another meta-analysis [60] examined the risk of infant mortality and short-term exposure to ambient air pollutants including PM_10_, O_3_, NO_2_, CO, and SO_2_. The finding showed unclear and contradictory associations, with limited-suggestive evidence (**Figure 3**, Table S11 in the **Online Supplementary Document**).

### Study overlap

In this umbrella review, a total of 719 primary studies were reported. We conducted a study overlap analysis for each association reported by multiple meta-analyses, The analysis indicated a slight overlap among primary studies included in the meta-analyses, with a CCA ranging from zero to 0.2% (Table S12 in the **Online Supplementary Document**).

### Heterogeneity and publication bias

Among 27 meta-analyses identified in the umbrella review, five [24,54,58,59,66] did not report the level of heterogeneity among the included studies. Of the reported associations for U5M, we observed high heterogeneity for factors such as maternal death [33], short IPIs [23], PM_2.5_, and PM_10_ [49]. Furthermore, out of the total 52 associations reported for infant mortality, *I*^2^ values were provided for 43 observed associations. In nine of these associations, we identified substantial to high heterogeneity. However, heterogeneity analyses were not undertaken in most meta-analyses due to the small number of included studies.

Of the total meta-analyses, publication bias was assessed in 22 meta-analyses [23–25,27–29,31,33,34,49–53,56,57,60,61,63,64,66,67]. We observed significant publication bias in studies investigating the association of short IPIs [23] with U5M. Moreover, significant publication bias was reported for the association of short interpregnancy intervals [23,34], and short-term exposure to NO_2_ and CO [60] with infant mortality. Trim and fill analyses found similar findings with the initial results except for NO_2_ [60]. However, it is important to note that 40 associations related to infant mortality were based on fewer than 10 studies, which limited the assessment of publication bias and the reliability of the results (Tables S10–11 in the **Online Supplementary Document**).

## DISCUSSION

This umbrella review represents the first comprehensive synthesis of risk factors associated with U5M and infant mortality, drawing from 32 systematic reviews and meta-analyses. We identified 28 unique factors associated with U5M and 29 unique factors associated with infant mortality, covering sociodemographic and economic, reproductive health-related and maternal death, child-related and environmental factors. Despite significant global attention and efforts aimed at addressing U5M, including initiatives by international health organisations and the SDGs [6], we did not find a convincing level of evidence supporting the identified risk factors. We found probable evidence of association between exclusive breastfeeding (consistent negative association), and maternal death (consistent positive association) with U5M. There was probable evidence for the association of short IPIs (less consistent negative association), pre-pregnancy maternal obesity (consistent positive association), and maternal HIV infection (consistent positive association) with infant mortality.

A meta-analysis [52] on the impact of breastfeeding on under-five mortality showed a consistent negative association between EBF and U5M. Children exclusively breastfed for the first six months had an 82% lower risk of U5M [52], which is similar to findings from a previous overview on breastfeeding [68]. However, the magnitude of reduction in U5M should be interpreted cautiously as the included studies mainly focused on mortality during the neonatal period and infancy. Furthermore, the applicability of these findings to high-income settings may be limited, given that the meta-analysis was conducted in sub-Saharan Africa. Exclusive breastfeeding may reduce U5M by providing infants with essential nutrients, enhancing immune protection, promoting optimal growth and development, and supporting digestive health, thereby lowering the risk of vulnerability to infections and malnutrition [69–71]. This is particularly important in LMICs, where the majority of under-5 deaths occur [3]. Optimal breastfeeding is estimated to avert 820 000 under-5 deaths annually [68]. Despite this, the global rate of EBF remains low, with approximately 56% of infants not receiving EBF [72]. This review provides evidence for investing in EBF to enhance child health and survival, but more research, mainly from high-income countries is required.

Maternal death is a risk factor for U5M, showing probable evidence of consistent positive association [33]. However, the evidence is based on studies from LMICs, which limits its generalisability to high-income countries (HICs). While the exact mechanisms are not yet fully established, the loss of a maternal caregiver deprives children of essential nurturing and breastfeeding, increasing the risk of inadequate nutrition [73]. Disruption of breastfeeding and premature weaning [74] may also increase children’s susceptibility to infection and malnutrition. Moreover, children who lose their mothers may face barriers to accessing child health care services [75] due to financial strain and psychosocial stress within the family.

Maternal and paternal education [24] were identified as protective factors against U5M, supported by a previous overview [76]. The association showed a dose-response relationship, with mothers who completed primary education having a 16% reduced risk of U5M, while those with secondary and tertiary education had even greater reductions of 31 and 39%, respectively. Furthermore, paternal education showed a similar pattern but with slightly lower effects. Children of primary-educated fathers had a 9% reduced risk of U5M, while those with fathers who attained secondary and tertiary education experienced even more reductions, with risks lowered by 17 and 22%, respectively. Though not graded, the evidence was supported by a substantial number of studies globally. Education enhances skill building and socioeconomic status, increasing access to information, delaying childbearing, improving maternal and child health service utilisation, as well as promoting maternal health and optimal birth spacing [77,78]. These findings support the need to increase investment in inclusive and equitable quality education as part of the SDG agenda.

Short IPI was examined in four systematic reviews [23,30,34,59], three of which included meta-analyses, yielding diverse findings. A meta-analysis [59] with probable evidence indicated an increased risk of infant mortality among short IPIs of less than 18 months, compared to longer intervals (36 to 60 months) in LMICs. Two additional meta-analyses [23,34] conducted in LMICs, found a 2-fold increased risk of infant mortality, compared to intervals greater than 24 months, in line with WHO recommendations of waiting at least 24 months after a live birth before attempting the next pregnancy. However, the evidence was limited-suggestive, mainly based on cross-sectional studies. In HICs, a systematic review without meta-analysis [30] revealed an increased risk of infant mortality for intervals shorter than six months, compared to 18 to 23 months. Nevertheless, the available evidence remains scarce and warrants further investigation to strengthen the conclusions. Increased infant mortality may be attributed to adverse birth outcomes such as birth defects [79] and low birth weight [80,81] due to maternal nutritional depletion from short intervals [82]. Short IPIs can lead to preterm birth [59,81] due to maternal stress [83], and insufficient time for uterine involution and recovery from any existing endometritis.[84] Short IPIs may also interfere with breastfeeding, and contribute to infections [85] and malnutrition [86]. These findings underscore the importance of promoting adequate birth spacing to decrease child mortality in LMICs. However, further well-designed studies are needed to elucidate the specific IPIs associated with increased risk of infant mortality in different income settings and the underlying factors contributing to these disparities.

Maternal pre-pregnancy obesity showed a consistent positive association with infant mortality, supported by probable evidence [56,62]. The associations showed a dose-response relationship, with higher risks observed across different body mass index categories. Overweight women have an increased risk compared to those healthy weight, followed by even higher risks among class I obese women, and the greatest higher risks among class II obese women. Obesity increases the risk of gestational diabetes mellitus, hypertension, and preeclampsia, leading to adverse birth outcomes such as preterm birth, low birth weight, macrosomia, birth injuries, and respiratory distress syndrome [87,88]. Obesity may also induce chronic inflammation and oxidative stress which compromise placental function and nutrient supply to the foetus, resulting in foetal growth restriction and preterm birth. Metabolic dysfunction, hormonal imbalances, and epigenetic alterations associated with obesity may further contribute to birth defects or congenital anomalies [88,89]. However, most evidence comes from studies conducted in HICs, necessitating further research to determine the risk in other populations.

HIV-infected mothers face a 3-fold higher risk of infant mortality compared to non-infected mothers [54]. This increased risk can be attributed to several potential mechanisms. First, maternal HIV infection results in vertical transmission of HIV to infants, [90] which leads to a high risk of infant mortality due to the direct effects of HIV on their immune system and subsequent opportunistic infections [91]. Maternal HIV infection can also induce immune dysfunction in both mother and infant, increasing vulnerability to such infections. [92,93] Moreover, maternal HIV is associated with adverse birth outcomes such as preterm birth, and low birth weight [94,95], which are the main causes of infant mortality [96]. Additionally, sub-optimal breastfeeding practices related to maternal HIV can contribute to malnutrition and increased susceptibility to infections, further amplifying mortality risks among affected infants [97]. However, this risk should be interpreted cautiously as the included primary studies in this meta-analysis were conducted from 1988 to 1995 before the adoption and implementation of mother-to-child transmission services. Significant progress has been made since then in reducing HIV infection among children [98]. Nevertheless, a meta-analysis [53] conducted in 2016 showed a 70% increased risk of infant mortality among HIV-exposed uninfected infants compared to HIV-unexposed uninfected infants. Nevertheless, the evidence was rated as limited-suggestive, as the overall direction of the association is unclear or contradictory. Therefore, further investigation is warranted to strengthen the evidence.

To our knowledge, this is the first umbrella review that offers a comprehensive analysis, evaluation, and summary of the epidemiological evidence on risk factors of U5M and infant mortality. We conducted an extensive search across major databases, systematic review repositories, grey literature sources as well as references cited in the included reviews.

However, this review’s limitations should be considered when interpreting the findings. First, most included reviews were rated as having low and critically low quality according to AMSTAR 2 tool. This may raise concerns about the accuracy of the conclusion drawn and undermine the overall strength of the evidence. Second, most associations relied on a small number of studies, with the majority (n = 47) including less than ten primary studies, which compromises the generalisability of the findings, despite the large geographical coverage in many reviews. Third, incomplete information in some meta-analyses limited our ability to accurately grade the evidence for certain risk factors. Fourth, the majority of the associations were graded as limited-suggestive and limited no conclusive evidence due to limitations in study design and a small number of primary studies, highlighting the need for further well-designed research. Fifth, the high heterogeneity observed in some meta-analyses introduces uncertainty in our findings. The potential impact of publication bias should be considered, as it was not assessed in some meta-analyses. Additionally, inconsistencies in categorising factors such as maternal education, paternal education, and IPIs across systematic reviews hinder the ability to draw strong conclusions. Moreover, variability in confounder adjustment across the primary studies may introduce bias in the pooled analyses, further limiting the validity of our findings. The grading approach used in this review also has limitations in objectivity and entails a potential for mis-grading. Additionally, a systematic review [26] without meta-analysis provides limited clarity on the direction of the association and specific factor categories influencing U5M and infant mortality, thereby hindering the drawing of conclusive findings on these associations. Lastly, our review was limited to English language publications, raising concerns about the potential impact of non-English reviews on overall findings, though evidence indicated that non-English language exclusion had minimal effect on the overall conclusions [99,100]. Future studies that address these methodological shortcomings will be crucial for informing more robust and reliable conclusions.

## CONCLUSIONS

This umbrella review identified 28 and 29 unique factors associated with U5M and infant mortality, respectively. However, there is no convincing evidence on these risk factors. We found probable evidence of associations between exclusive breastfeeding (consistent negative association) and maternal death (consistent positive association) with U5M. Additionally, short IPIs (<18 months) (less consistent positive association), maternal obesity (consistent positive association), and maternal HIV (consistent positive association) are risk factors for infant mortality supported by probable evidence. Evidence for the remaining risk factors is limited-suggestive, or limited and non-conclusive. Further high-quality studies are required to strengthen the evidence on these factors.

## Additional Material


Online Supplementary Document
