# Supplementary material for: Risk factors of under-five and infant mortality: An umbrella review of systematic reviews and meta-analyses
Source: J Glob Health. 2024 Nov 29;14:04260. doi: 10.7189/jogh.14.04260 (PMC11605776; doi:10.7189/jogh.14.04260)
Supplement: Online Supplementary Document [file jogh-14-04260-s001.pdf]

Risk factors of under-five and infant mortality: an umbrella review of systematic reviews and meta-analyses

Bereket Kefale[1,2], Jonine Jancey[1,3], Amanuel T Gebremedhin[1 4], Sylvester Dodzi Nyadanu[1], Daniel G Belay[1], Gavin Pereira[1,3], Gizachew A Tessema[1,3,5]

[1] Curtin School of Population Health, Curtin University, Perth, WA, Australia

[2] Department of Reproductive Health, School of Public Health, Wollo University, Dessie, Ethiopia

[3]enAble Institute, Curtin University, Perth, WA, Australia

[4]School of Nursing and Midwifery, Edith Cowan University, Perth, WA, Australia

[5]School of Public Health, University of Adelaide, Adelaide, SA, Australia

Supplementary tables

Table S1. PRISMA 2020 Checklist
Table S2. Search strategy for an umbrella review on risk factors of under-five mortality and infant mortality
Table S3. Direction of association grading criteria
Table S4. Evidence grading criteria
Table S5. Formula used for converting OR to RR
Table S6. Standardising RR for air pollutants
Table S7. Excluded reviews and reasons for exclusion after full-text review

Table S8. Characteristics of systematic reviews and meta-analyses
Table S9. Summary results from systematic reviews and meta-analyses
Table S10. Summary of evidence of meta-analyses on under-five mortality.
Table S11. Summary of evidence of meta-analyses on infant mortality
Table S12. Study overlaps in systematic reviews

Supplementary figures

Figure S1. Quality assessment using AMSTAR 2 tool.
Figure S2. Publication date ranges of systematic reviews

Table S1. PRISMA 2020 Checklist

Section and Topic	Item #	Checklist item	Reported on page #
**TITLE**			
Title	1	Identify the report as a systematic review.	1
**ABSTRACT**			
Abstract	2	See the PRISMA 2020 for Abstracts checklist.	2
**INTRODUCTION**			
Rationale	3	Describe the rationale for the review in the context of existing knowledge.	2&3
Objectives	4	Provide an explicit statement of the objective(s) or question(s) the review addresses.	3
**METHODS**			
Eligibility criteria	5	Specify the inclusion and exclusion criteria for the review and how studies were grouped for the syntheses.	4
Information sources	6	Specify all databases, registers, websites, organisations, reference lists and other sources searched or consulted to identify studies. Specify the date when each source was last searched or consulted.	4
Search strategy	7	Present the full search strategies for all databases, registers and websites, including any filters and limits used.	4

Section and Topic	Item #	Checklist item	Reported on page #
Selection process	8	Specify the methods used to decide whether a study met the inclusion criteria of the review, including how many reviewers screened each record and each report retrieved, whether they worked independently, and if applicable, details of automation tools used in the process.	4
Data collection process	9	Specify the methods used to collect data from reports, including how many reviewers collected data from each report, whether they worked independently, any processes for obtaining or confirming data from study investigators, and if applicable, details of automation tools used in the process.	4-5
Data items	10a	List and define all outcomes for which data were sought. Specify whether all results that were compatible with each outcome domain in each study were sought (e.g. for all measures, time points, analyses), and if not, the methods used to decide which results to collect.	5
	10b	List and define all other variables for which data were sought (e.g. participant and intervention characteristics, funding sources). Describe any assumptions made about any missing or unclear information.	5
Study risk of bias assessment	11	Specify the methods used to assess risk of bias in the included studies, including details of the tool(s) used, how many reviewers assessed each study and whether they worked independently, and if applicable, details of automation tools used in the process.	5
Effect measures	12	Specify for each outcome the effect measure(s) (e.g. risk ratio, mean difference) used in the synthesis or presentation of results.	5
Synthesis methods	13a	Describe the processes used to decide which studies were eligible for each synthesis (e.g. tabulating the study intervention characteristics and comparing against the planned groups for each synthesis (item #5)).	5
	13b	Describe any methods required to prepare the data for presentation or synthesis, such as handling of missing summary statistics, or data conversions.	5
	13c	Describe any methods used to tabulate or visually display results of individual studies and syntheses.	5
	13d	Describe any methods used to synthesize results and provide a rationale for the choice(s). If meta-analysis was performed, describe the model(s), method(s) to identify the presence and extent of statistical heterogeneity, and software package(s) used.	5
	13e	Describe any methods used to explore possible causes of heterogeneity among study results (e.g. subgroup analysis, meta-regression).	N/A
	13f	Describe any sensitivity analyses conducted to assess robustness of the synthesized results.	N/A
Reporting bias assessment	14	Describe any methods used to assess risk of bias due to missing results in a synthesis (arising from reporting biases).	N/A
Certainty assessment	15	Describe any methods used to assess certainty (or confidence) in the body of evidence for an outcome.	5
**RESULTS**			
Study selection	16a	Describe the results of the search and selection process, from the number of records identified in the search to the number of studies included in the review, ideally using a flow diagram.	5
	16b	Cite studies that might appear to meet the inclusion criteria, but which were excluded, and explain why they were excluded.	5
Study	17	Cite each included study and present its characteristics.	5

Section and Topic	Item #	Checklist item	Reported on page #
characteristics			
Risk of bias in studies	18	Present assessments of risk of bias for each included study.	5-6
Results of individual studies	19	For all outcomes, present, for each study: (a) summary statistics for each group (where appropriate) and (b) an effect estimate and its precision (e.g. confidence/credible interval), ideally using structured tables or plots.	Page 5, Table 1, S2 Table
Results of syntheses	20a	For each synthesis, briefly summarise the characteristics and risk of bias among contributing studies.	5-6,
	20b	Present results of all statistical syntheses conducted. If meta-analysis was done, present for each the summary estimate and its precision (e.g. confidence/credible interval) and measures of statistical heterogeneity. If comparing groups, describe the direction of the effect.	6-12, S4-S5 Tables
	20c	Present results of all investigations of possible causes of heterogeneity among study results.	11, S4, S5 Tables
	20d	Present results of all sensitivity analyses conducted to assess the robustness of the synthesized results.	N/A
Reporting biases	21	Present assessments of risk of bias due to missing results (arising from reporting biases) for each synthesis assessed.	N/A
Certainty of evidence	22	Present assessments of certainty (or confidence) in the body of evidence for each outcome assessed.	6-11, Supplementary Table 8-9
**DISCUSSION**			
Discussion	23a	Provide a general interpretation of the results in the context of other evidence.	12-16
	23b	Discuss any limitations of the evidence included in the review.	15-16
	23c	Discuss any limitations of the review processes used.	15-16
	23d	Discuss implications of the results for practice, policy, and future research.	12-16
**OTHER INFORMATION**			
Registration and protocol	24	Provide registration information for the review, including register name and registration number, or state that the review was not registered.	2, 4
Support	25	Describe sources of financial or non-financial support for the review, and the role of the funders or sponsors in the review.	11
Competing interests	26	Declare any competing interests of review authors.	11
Availability of data, code and other materials	27	Report which of the following are publicly available and where they can be found: template data collection forms; data extracted from included studies; data used for all analyses; analytic code; any other materials used in the review.	11

Table S2. Search strategy for an umbrella review on risk factors of under-five mortality and infant mortality

	Concept 1	Concept 2	Concept 3
	**Risk factors**	**Under-five mortality**	**Systematic review and meta-analysis**
**Keywords (Ovid)**	("risk factor*" or "associated factor*" or "factor* associated" or determinant* or predictor* or factor* or effect* or associat*).ti,ab.	((infant or child* or under-five or under-5 or "under five" or "under 5" or baby or toddler* or paediatric*) ADJ2 (mortalit* or death*)).ti,ab.	("systematic review*"or meta-analy* or metaanaly*).ti,ab.
**Embase headings**	exp risk factor/	exp infant mortality/ or exp child death/ or exp childhood mortality/	exp "systematic review"/ or exp meta analysis/ or exp "systematic review (topic)"/ or exp "meta analysis (topic)"/
**Medline headings**	exp Risk Factors/	exp Infant Mortality/ OR exp Child Mortality/	exp "systematic review"/ or exp meta-analysis/ or exp "Systematic Reviews as Topic"/ or exp "Meta-Analysis as Topic"/
**Keywords (EBSCO)**	TI ("risk factor*" or "associated factor*" or "factor* associated" or determinant* or predictor* or factor* or effect* or associat*) or AB ("risk factor*" or "associated factor*" or "factor* associated" or determinant* or predictor* or factor* or effect* or associat*)	TI ((infant or child* or under-five or under-5 or "under five" or "under 5" or baby or toddler* or paediatric*) N2 (mortalit* or death*)) OR AB ((infant or child* or under-five or under-5 or "under five" or "under 5" or baby or toddler* or paediatric*) N2 (mortalit* or death*))	TI (systematic review* or meta-analy*) or AB (systematic review* or meta-analy*)
**CINAHL headings**	(MH "Risk Factors+")	(MH "Perinatal Death") OR (MH "Infant Mortality") OR (MH "Child Mortality")	(MH "Systematic Review") OR (MH "Meta Analysis")
**Keywords Scopus/Web of science**	("risk factor*" or "associated factor*" or "factor* associated" or determinant* or predictor* or factor* or effect* or associat*)	("infant death*" or "child* death*" or "under-five death*"or "under-5 death*"or "under five death*" or "under 5 death*" or "baby death*" or "toddler* death*" or "paediatric* death*" or "infant mortalit*" or "child* mortalit*" or "under-five mortalit*" or "under-5 mortalit*"or "under five mortalit*" or "under 5 mortalit*" or "baby death*" or "toddler* mortalit*" or "paediatric* mortalit*")	(systematic review* or meta-analy* or metaanaly*)

Searching strategies for each database

Databases	s.no	Headings, key terms with proximity operators
**Embase (Ovid)**	#1	exp risk factor/
	#2	("risk factor*" or "associated factor*" or "factor* associated" or determinant* or predictor* or factor* or effect* or associat*).ti,ab.
	#3	#1 or #2
	#4	exp infant mortality/ or exp child death/ or exp childhood mortality/
	#5	((infant or child* or under-five or under-5 or "under five" or "under 5" or baby or toddler* or paediatric*) ADJ2 (mortalit* or death*)).ti,ab.
	#6	#4 or #5
	#7	exp "systematic review"/ or exp meta analysis/ or exp "systematic review (topic)"/ or exp "meta analysis (topic)"/
	#8	("systematic review*"or meta-analy* or metaanaly*).ti,ab.
	#9	#7 or #8
	#10	#3 and #6 and #9
	#11	limit (English language and yr="1990 -Current") and (human) and (article or article in press or "preprint)
**Medline (Ovid)**	#1	exp risk factors/
	#2	("risk factor*" or "associated factor*" or "factor* associated" or determinant* or predictor* or factor* or effect* or associat*).ti,ab.
	#3	#1 or #2
	#4	exp Infant Mortality/ or exp Infant Death/ or exp Child Mortality/
	#5	((infant or child* or under-five or under-5 or "under five" or "under 5" or baby or toddler* or paediatric*) ADJ2 (mortalit* or death*)).ti,ab.
	#6	#4 or #5
	#7	exp "systematic review"/ or exp meta-analysis/ or exp "Systematic Reviews as Topic"/ or exp "Meta-Analysis as Topic"/
	#8	("systematic review*"or meta-analy* or metaanaly*).ti,ab.
	#9	#7 or #8
	#10	#3 and #6 and #9
	#11	Limit (English language and yr="1990 -Current") and (human) and (journal article or "preprint)

**Global Health (Ovid)**	#1	exp risk factors/
	#2	("risk factor*" or "associated factor*" or "factor* associated" or determinant* or predictor* or factor* or effect* or associat*).ti,ab.
	#3	#1 or #2
	#4	exp infant mortality/
	#5	((infant or child* or under-five or under-5 or "under five" or "under 5" or baby or toddler* or paediatric*) ADJ2 (mortalit* or death*)).ti,ab.
	#6	#4 or #5
	#7	exp systematic reviews/ or exp meta-analysis/
	#8	("systematic review*"or meta-analy* or metaanaly*).ti,ab.
	#9	#7 or #8
	#10	#3 and #6 and #9
	#11	Limit (English language and yr="1990 -Current" and journal article or preprint or theis)
**Scopus**	#1	TITLE-ABS-KEY ("risk factor*" or "associated factor*" or "factor* associated" or determinant* or predictor* or factor* or effect* or associat*)
	#2	TITLE-ABS-KEY ("infant death*" OR "child* death*" OR "under-five death*" OR "under-5 death*" OR "under five death*" OR "under 5 death*" OR "baby death*" OR "toddler* death*" OR "paediatric* death*" OR "infant mortalit*" OR "child* mortalit*" OR "under-five mortalit*" OR "under-5 mortalit*" OR "under five mortalit*" OR "under 5 mortalit*" OR "baby death*" OR "toddler* mortalit*" OR "paediatric* mortalit*")
	# 3	TITLE-ABS-KEY ("systematic review*" or meta-analy* or metaanaly*)
	# 4	#1 and #2 and #3
	# 5	Pubyear > 1990 and pubyear < 2024 and (limit-to (language, "English") and document type- article
**Web of science**	#1	TI=(("risk factor*" or "associated factor*" or "factor* associated" or determinant* or predictor* or factor* or effect* or associat*)) OR AB=(("risk factor*" or "associated factor*" or "factor* associated" or determinant* or predictor* or factor* or effect* or associat*))
	#2	TI=(("infant death*" or "child* death*" or "under-five death*"or "under-5 death*"or "under five death*" or "under 5 death*" or "baby death*" or "toddler* death*" or "paediatric* death*" or "infant mortalit*" or "child* mortalit*" or "under-five mortalit*" or "under-5 mortalit*"or "under five mortalit*" or "under 5 mortalit*" or "baby death*" or "toddler* mortalit*" or "paediatric* mortalit*")) OR AB=(("infant death*" or

		"child* death*" or "under-five death*"or "under-5 death*"or "under five death*" or "under 5 death*" or "baby death*" or "toddler* death*" or "paediatric* death*" or "infant mortalit*" or "child* mortalit*" or "under-five mortalit*" or "under-5 mortalit*"or "under five mortalit*" or "under 5 mortalit*" or "baby death*" or "toddler* mortalit*" or "paediatric* mortalit*"))
	#3	TI=(("systematic review*" or meta-analy* or metaanaly*)) OR AB=(("systematic review*" or meta-analy* or metaanaly*))
	#4	#1 and #2 and #3
	#5	Limited to English, review article and article
**CINAHL (EBSCO)**	#1	(MH "Risk Factors+")
	#2	TI ("risk factor*" or "associated factor*" or "factor* associated" or determinant* or predictor* or factor* or effect* or associat*) or AB ("risk factor*" or "associated factor*" or "factor* associated" or determinant* or predictor* or factor* or effect* or associat*)
	#3	#1 or #2
	#4	(MH "Infant Mortality") OR (MH "Child Mortality")
	#5	TI ((infant or child* or under-five or under-5 or "under five" or "under 5" or baby or toddler* or paediatric*) N2 (mortalit* or death*)) OR AB ((infant or child* or under-five or under-5 or "under five" or "under 5" or baby or toddler* or paediatric*) N2 (mortalit* or death*))
	#6	#4 or #5
	#7	(MH "Systematic Review") OR (MH "Meta Analysis")
	#8	TI (systematic review* or meta-analy* or metanaly*) or AB (systematic review* or meta-analy* or meta-analy*)
	#9	#7 or #8
	#10	#3 and #6 and #9
	#11	Limiters - Published Date: 1990-2023, and Language: - English
**Cochrane Database of Systematic Reviews**	#1	("risk factor" or "associated factor" or "factor associated" or determinant or predictor or factor or effect or association):ti,ab,kw - (Word variations have been searched)
	#2	("infant death" OR "child death" OR "under-five death" OR "under-5 death" OR "under five death" OR "under 5 death" OR "baby death" OR "toddler death" OR "paediatric death" OR "infant mortality" OR "child mortality" OR "under-five mortality" OR "under-5 mortality" OR "under five mortality" OR "under 5 mortality" OR "baby death" OR "toddler mortality" OR "paediatric mortality") ti,ab,kw - (Word variations have been searched)
	#3	#1 and #2
	#4	Filter: year: since 1990, Cochrane review
**Epistemonikos**	#1	(title:((title:("risk factor*" OR "associated factor*" OR "factor* associated" OR determinant* OR predictor* OR factor* OR effect* OR associate*) OR abstract:("risk factor*" OR "associated factor*" OR "factor* associated" OR determinant* OR predictor* OR factor* OR effect* OR associate*))) OR abstract:((title:("risk factor*" OR "associated factor*" OR "factor* associated" OR determinant* OR

		predictor* OR factor* OR effect* OR associat) OR abstract:("risk factor*" OR "associated factor*" OR "factor* associated" OR determinant* OR predictor* OR factor* OR effect* OR associat))))
	#2	(title:((title:("infant* death*" OR "infant* mortalit*" OR "child* death*" OR "child* mortalit*" OR "toddler* death*" OR "toddler* mortalit*" OR "under-five death*" OR "under-five mortalit*" OR "under-five child* death*" OR "under-5 death*" "under-5 mortalit*" OR "under-five child* mortalit*" OR "death* in child*" OR "mortalit* in child*") OR abstract:("infant* death*" OR "infant* mortalit*" OR "child* death*" OR "child* mortalit*" OR "toddler* death*" OR "toddler* mortalit*" OR "under-five death*" OR "under-five mortalit*" OR "under-five child* death*" OR "under-5 death*" "under-5 mortalit*" OR "under-five child* mortalit*" OR "death* in child*" OR "mortalit* in child*")))
	#3	#1 and #2
	#4	Publication date since 1990, Publication type: Systematic review
**Google Scholar** (first 200 hits where available)		("risk factors"|"associated factors"|determinants|predictors) ("infant mortality"|"child death"|"child mortality"|"under-five death"|"under-five mortality") ("systematic review"| meta-analysis|meta-analyses)

Table S3. Direction of association grading criteria

Category on importance of risk factors	Grading criteria
"++" (Consistent positive association)	The variable shows a positive association in at least 80% of reviews. It also means that either a single review included the variable and demonstrated a significant positive association and/or reported (non)-significant effect sizes (RR, HR) larger than 1.5, in at least 80% of included primary studies.
"+" (Less consistent positive association)	The variable shows a positive association in at least 60% of reviews. It also means that either a single review included the variable and demonstrated a significant positive association and/or reported (non)-significant effect sizes (RR, HR) larger than 1.5, in at least 60% of included primary studies.
"00" (Consistent null association)	The variable shows a null association in at least 80% of reviews. It also means that either a single review included the variable and demonstrated a null association in at least 80% of included primary studies.
"0" (Less consistent null association)	The variable shows a null association in at least 60% of reviews. It also means that either a single review included the variable and demonstrated a null association in at least 60% of included primary studies.
"?" (Unclear or contradictory)	If there is no agreement in the direction of association for at least 60% of reviews, or included primary studies, the direction of association is graded as unclear or contradictory.
"-" (Less consistent negative association)	The variable shows a negative association in at least 60% of reviews. It also means that either a single review included the variable and demonstrated a significant negative association and/or reported (non)-significant effect sizes (RR, HR) lower than 0.66 in at least 60% of included primary studies.
-- (Consistent negative association)	The variable shows a negative association in 80% of reviews. It also means that either a single review included the variable and demonstrated a significant negative association and/or reported (non)-significant effect sizes (RR, HR) lower than 0.66 in at least 80% of included primary studies.

Table S4. Evidence grading criteria

**Strength of evidence**	**Grading criteria**
Convincing evidence (Ce)	The evidence should be based on studies of risk factors showing a consistent association between the variable and outcome. The available evidence is based on a substantial number of studies, including longitudinal observation studies and where relevant, experimental studies of sufficient size, duration, and quality showing consistent effects. Specifically, the grading criteria require evidence from multiple study types, with at least two independent cohort studies available, as well as strong and plausible experimental evidence.
Probable evidence (Pe)	The evidence should be based on studies of risk factors showing fairly consistent associations between the variable and the outcome, but there may be shortcomings in the available evidence or some evidence to the contrary, which precludes a more definite judgment. Shortcomings in the evidence may be any of the following: insufficient duration of studies, insufficient studies available, inadequate sample sizes, and incomplete follow-up. However, evidence from at least two independent cohort studies or five case-control studies should be available.
Limited suggestive evidence (Ls)	Evidence is based mainly on findings of cross-sectional studies. Insufficient longitudinal observational studies or experimental studies are available, or results are inconsistent. More well-designed studies of risk factors are required to support the tentative associations.
Limited, no conclusive evidence (Lnc)	Evidence is based on findings of a few studies which are suggestive but are insufficient to establish an association between the variable and the outcome. No evidence is available from longitudinal observational or experimental studies. More well-designed studies are needed to support the tentative association.

Table S5. Formula used for converting OR to RR

Formula for converting OR to RR	$RR=\frac{OR}{1+p(OR-1)}$<br>where p is the prevalence among the control population.[1]

Table S6. Standardising RR for air pollutants

Standardising RR for air pollutants	We standardised the RR across reviews to a 10 μg/m^3 increase in air all pollutants concentrations (PM_{10}, $PM_{2.5}$, SO_2, NO_2, O_3) and 1 mg/m^3 for CO. The reported review result in ppb or ppm, were converted to the standardised measures as follows.[2]<br><br>NO_2 (1ppb) = 1.88 μg/ m3<br><br>SO2 (1ppb) = 2.62 μg/m3<br><br>O_3 (1 ppb) = 2.00 μg/m3<br><br>CO (1 ppm= 1000 ppb) = 1.145 mg/m3.<br><br>1 mg/m3 = 1000 μg/m3<br><br>Then<br><br>Standardised increment<br><br>RR (standardised)= $RR(original)^{\left(\frac{Standardised\ increment}{Original\ increment}\right)}$

Table S7. Excluded reviews and reasons for exclusion after full-text review

S.N	Reviews	Reasons
1	Adinew 2017[3]	The result on risk factors of U5M was based on literature review, not systematic review.
2	Bede-Ojimadu 2020[4]	The outcome "under-five mortality" was reported by only a single study included primary study.
3	Doku 2020[5]	This is individual participant meta-analysis, not a systematic review with meta-analysis.
4	Gissler 2009[6]	The finding for infant mortality was not clear. It was mixed with the finding of stillbirth.
5	Islam 2021[7]	This is individual participant meta-analysis, not a systematic review with meta-analysis
6	Islam 2022[8]	Duplication
7	Khan 1999[9]	This is a commentary.
8	Kihal-Talantikite 2020[10]	Duplication
9	Kihal-Talantikite 2020[10]	The outcome was postnatal mortality, not infant mortality.
10	Kim 2013[11]	This review included systematic reviews, not only primary studies.
11	Lacasana 2005[12]	The outcome was adverse birth outcomes and early neonatal death, not infant mortality.

12	Lamberti 2013[13]	The outcome of the review is a cause specific mortality not total mortality status.
13	McDonald 2013[14]	This is individual participant meta-analysis, not a systematic review with meta-analysis.
14	Musyoka 2021[15]	The meta-analyses were conducted wrongly, by merging negative and positive associations
15	O'Hare 2013[16]	The estimate is income elasticity, not RR, OR, or β.
16	Ortigoza 2017[17]	This article is not a systematic review.
17	Pillai 1997[18]	This is individual participant meta-analysis, not a systematic review with meta-analysis.
18	Purssell 2015[19]	The outcome is fever phobia, not under-five mortality.
19	Rowe 2021[20]	The review reported an increase in the proportion infant mortality but did not examine the association.
20.	Rutherford 2010[21]	This is literature review, not a systematic review.
21	Sankar 2015[22]	Duplication
22	Shi 2022[23]	The study population were children with syncytial virus-associated respiratory tract infection, not general population.
23	Victora 2000[24]	The review's outcome was infectious disease related mortality, but not all cause mortality.
24	Wendt 2012[25]	The outcomes were adverse pregnancy outcomes and early neonatal outcome, not infant mortality.
25	Wigle 2008[26]	This is literature review, not a systematic review
26	Xu 2014[27]	The outcome "infant mortality" was reported by a single study.

Table S8. Characteristics of systematic reviews and meta-analyses

**First author, (No. of authors, countries)**	**Risk/protective factors**	**Outcomes**	**No. of databases, grey literature**	**Search date range and language applied**	**No. of primary studies, study design, coverage**	**No. of eligible studies included in the UR; study design**	**Publication year range**	**Total no. of participants; no. of participants in eligible studies**	**Risk of bias assessment tool**	**Reporting guideline**	**Evidence of pre-specified protocol**	**Quality of reviews**
Balaj 2021[28] (19; 10 USA, 9 Norway)	Maternal education, paternal education	U5M	DB=5 GL=Yes	Inception - January 2021. No language restriction	300; unclear. Global	300; unclear.	1982-2020	Unclear, > 3,112,474 live births	NA	PRISMA	Yes	Moderate

Bhusal 2022 [29] (2; all Nepal	Socio-demographic and economic factors, maternal reproductive health related factors, child related factors	U5M	DB=4 GL=Yes	Inception - August 10, 2022. English	22; 1 cohort, 21 cross-sectional. Global	22; 1 cohort, 21 cross-sectional.	2006-2022	>413,551 participants (1 study not reported)	National Institute of Health quality assessment tools	PRISMA	No	Critically low
Chikhungu 2017[30] (3; 2 England, 1 Switzerland)	Maternal death	U5M	DB=5 GL=Yes	1st January 1990 – 30th November 2016. English	7;1 Randomized controlled trial, 4 cohort, 2 Birth and death registers. Global	7; 1 Randomized controlled trial, 4 cohort, 2 Birth and death registers.	2002-2013	190,859 children; 152,201 children	GRADE	PRISMA	No	Low
Forde 2017[31] (2; all Trinidad and Tobago)	Residence	U5M	DB=3 GL=Yes	1990 - 2015, English, Spanish	26; all cross-sectional LMICs	26; all cross-sectional	2001-2014	NA	NA	NA	No	Critically low
Garoma 2011 [32] (3; all Ethiopia)	Intimate partner violence against women	U5M	DB=4 GL=Yes	Inception - December 2010. English	11; 3 cohort, 2 case control, 6 cross-sectional. Global	11; 3 cohort, 2 case control, 6 cross-sectional.	2003-2010	87,394 women	NA	NA	No	Critically low

Huang 2017 [33] (6; China)	Ethnicity	U5M	DB=6 GL=No	January 1st, 1990, - November 9th, 2016. English, Chinese	31; 28 Cross-sectional, 3 routine health data report. Western China	2; all cross-sectional	1996 - 2015	6,492,366 participants;10,075 participants	The component approach adopted by the Cochrane Collaboration	PRISMA	No	Low
Karimi 2020 [34] (2; Iran)	Air pollution	U5M	DB=3 GL=No	January 1st, 1992 - December 30th, 2018. No language restriction	27; 8 case-crossover, 5 cohorts, 4 case-controls, 4 time-series, 4 cross-sectiona1, 2 ecological. Global	27; 8 case-crossover, 5 cohorts, 4 case-controls, 4 time-series, 4 cross-sectiona1, 2 ecological.	1997-2018	Incomplete	The tool reported by the National Institutes of Environmental Health Sciences-National Toxicology program	PRISMA	Yes	Moderate
Pretorius 2020 [35] (5; 4 South Africa, 1 Germany)	Non-exclusive breastfeeding	U5M	DB=2 GL=No	01 January 2000 - 31 May 2019. No language restriction	16; 11 cohort, 3 case-control, 2 cross-sectional.<br><br>SSA	6; 4 cohort, 1 case-control, 1 cross-sectional	2000-2019	30,801 participants	NOS	PRISMA	No	Low
Islam 2022 [8] (7; 3 Bangladesh, 1 Malaysia, 1 Australia)	Short interpregnancy interval	Infant mortality, U5M	DB=8 GL=No	January 2000 - January 2022. English	51; 3 cohort, 3 case-control, 45 cross-sectional. LMICs	24; 1 case-control, 23 cross-sectional	2010-2021	>869,615 participants (2 studies didn't report); >814,783 (1 study not reported)	NOS	PRISMA, STROBE	No	Low

Karami 2024 [36] (3; all Iran)	Socio-demographic and economic factors, maternal reproductive health-related factors, child-related factors	Infant mortality, U5M	DB=5 GL=Yes	Inception - January 2022. English, Farsi	32: 8 case-control, 21 cross-sectional, 2 time-series, 1 ecological. Iran	32: 8 case-control, 21 cross-sectional, 2 time-series, 1 ecological.	2000-2022	NA	STROBE	PRISMA	No	Low
Adane 2021[37] (8; all Australia)	Any maternal mental illness, severe mental illness, maternal anxiety, and depression	Infant mortality	DB=4 GL=Yes	Inception-December 2019. English	28; 24 cohort, 2 case-control, 2 cross-sectional. Global	11; incomplete	1997-2020	78,749,252 participants; 6,861,091 participants	NOS	PRISMA and MOOSE	Yes	Moderate
Ahrens 2018[38] (5; 4 USA, 1 Canada)	Short interpregnancy intervals	Infant mortality	DB=5 GL=No	1 January 2006-1 May 2017. English	32; 31 cohort, 1 case-control. HICs	4; 3 cohort; 1 case-control	1988-2017	5,132,928 participants; 220, 676 participants	Criteria outlined by the US. Preventive Services Task Force	PRISMA	Yes	Moderate
Aune 2014[39] (4; 2 Norway, 1 UK, 1 USA)	Body mass index	Infant mortality	DB=2 GL=No	Inception - January 23, 2014. NA	38: all cohort. Global	4; all cohort	2001-2012	NA; 1,491, 879 participants	NOS	NA	No	Low

Bagade 2018[40] (5; all Australia)	Intimate partner violence against women	Infant mortality	DB=5 GL=Yes	2000-2017. English	3; all cross-sectional. Global	2; all cross-sectional.	2008-2014	54,414 participants	Effective Public Health Practice Project	PRISMA	Yes	Low
Brennan 2016[41] (11; 10 USA, 1 South Africa)	HIV exposure	Infant mortality	DB=5 GL=Yes	1 January 1994-1 April 2016. English	22; all cohort. Global	22; all cohort.	1994-2015	29,212 participants	NA	NA	No	Low
Brocklehurs 1998[42] (2; all UK)	Maternal HIV infection	Infant Mortality	DB=3 GL=No	1983 to December 1996. NA	31: all cohort. Global	9; All cohort	1988-1996	46,878 women; 6,836 women	A predefined set of criteria	NA	Yes	Low
Dadi 2015[43] (1; Ethiopia)	Short interpregnancy interval	Infant Mortality	DB=3 GL=Yes	January 1st, 2000 - September 1st, 2013. English	5; 1 cohort, 2 case-control, 2 cross-sectional. Ethiopia	5; 1 cohort, 2 case-control, 2 cross-sectional.	2010-2013	43,909 infants	NA	PRISMA	No	Critically low
Eltayib 2023[44] (5; all Oman)	Socio-demographic and economic factors, maternal reproductive health related factors, child related factors	Infant mortality	DB=12 GL=Yes	Inception - July 2022. English and Arabic	27; 10 cohort, 1 case-control, 16 cross-sectional. Gulf Cooperation Council countries	27; 10 cohort, 1 case-control, 16 cross-sectional.	1982-2021	340,508 participants	JBI	PRISMA	No	Low

Glinianaia 2004[45] (5; all UK)	Air pollution	Infant mortality	DB=14 GL=Yes	January 1st, 1966, - December 31st, 2003. English	15; 2; cohort, 1 case-control, 10 ecologic or time series, 2 cross-sectional. Global	8; NA.	1972-2003	> 13 million births (Incomplete)	NA	U.K. National Health Service Centre for Reviews and Dissemination	No	Critically low
Huo 2021[46] (7; 6 China, 1 Germany)	Maternal body mass index	Infant mortality	DB=3 GL=No	Inception - November 26th, 2020. No language restriction.	22; all cohort. Global	8; all cohort.	2001-2020	13,532,293 participants	NOS	MOOSE	No	Low
Jacques 2019[47] (5; all Brazil)	Postnatal depressive symptoms or depression	Infant mortality	DB=2 GL=No	Inception to September 2017, French, English, Spanish, Portuguese	6; all cohort. Global	3; all cohort.	2001-2015	170371 participants; 140,275 participants	Downs and Black quality index	PRISMA	No	Low
Jahan 2007[48] (1; Saudi Arabia	Poverty	Infant Mortality	DB=4 GL=No	Inception - February 2007. English	9; 2 case-control, 7 cross-sectional. Eastern Mediterranean region	9; 2 case-control, 7 cross-sectional	1992-2006	NA	NA	NA	No	Critically low

Kiross 2019[49] (4; 2 Ethiopia, 2 Australia)	Maternal educational status	Infant mortality	DB=5 GL=No	Inception to February 20, 2018. NA	5; 1 cohort, 1 case-control, 3 cross-sectional. Ethiopia	5; 1 cohort, 1 case-control,3 cross-sectional.	2000-2015	39,542 participants	Joanna Briggs Institute Meta-Analysis of Statistics Assessment and Review Instrument	PRISMA	No	Low
Kozuki 2013[50] (9; 6 USA, 2 Brazil, 1 Zimbabwe)	Birth interval	Infant mortality	DB=2 GL=Yes	NA	5; all cohort. LMICs	5; all cohort.	1982-2004	32,670 singleton live births	NA	NA	No	Critically low
Luben 2023[51] (7; all USA)	Air pollution	Infant mortality	DB=2 GL=No	Inception - May 2022. English	22; 10 Case-crossover, 7 time-series, 3 case-control, 2 cross-sectional. Global	22; 10 Case-crossover, 7 time-series, 3 case-control, 2 cross-sectional	1999-2021	NA	Tool adapted from modified Office of Health Assessment and Translation framework	NA	No	Low
Mazzone 2023[52] (6; all UK)	Maternal epilepsy	Infant mortality	DB=6 GL=Yes	Inception - December 6th, 2022. No language restriction	76; 66 cohort, 9 case-control, 1 cross-sectional. Global	13; NA	1972-2022	Incomplete; 1,426,692 pregnancies	NOS	PRISMA	Yes	Low

Meehan 2014[53] (5; all England)	Maternal body mass index	Infant mortality	DB=5 GL=Yes	1993 - April 2012. English	24; 23 cohort, 1 case-control. Global	24; 23 cohort, 1 case-control	1994-2012	>4,343,442 participants (4 studies did not reported)	NOS	PRISMA	Yes	Moderate
Nguyen 2019[54] (7; 5 Australia, 2 USA)	Maternal death	Infant mortality	DB=2 GL=Yes	January 1980 – March 2017. English	12; Incomplete. LMICs	2; 1 cohort, 1 not specified	2003-2015	702,112 participants; 37,853 participants	NA	PRISMA	No	Low
Quansah 2015[55] (11; 2 Finland, 6 Ghana, 2 Canada, 1 China)	Arsenic exposure	Infant mortality	DB=3 GL=Yes	1946 - July 2013. NA	23; 11 cohort, 2 case-control, 10 cross-sectional. Global	7; 5 cohort, 2 cross-sectional	1989-2013	>67,426 participants (1 study did not report: >43,616 participants	NOS	PRISMA	No	Low
Rahman 2021[56] (4; all India)	Residence	Infant mortality	DB=3 GL=No	2010 to 2015. NA	65; All cross-sectional. India	65; All cross-sectional.	2010-2015	NA	NA	NA	No	Critically low
Sankar 2015[57] (7; 5 India, 1 Norway, 1 Switzerland)	Breastfeeding	Infant mortality	DB=3 GL=No	Inception - September 2009. English	13; 9 cohort, 2 case-control, 2 secondary analysis of RCT. Global	3; 1 cohort, 2 secondary analysis of RCT	1987-2006	46,499 participants	Guideline Development Tool (GDT) developed by the GRADE Working Group	NA	No	Critically low
Weightman 2012[58] (6: all UK)	Area deprivation, social class	Infant mortality	DB=26 GL=Yes	1994 - May 2011. No language restriction	36; 30 cohort, 6 case-control. UK	4; all cohort	1994-2010, (2 unpublished)	Incomplete; 16,428,337 live births	NOS	NA	No	Low

DB, database; GL, grey literature; GRADE: Grading of Recommendations, Assessment, Development and Evaluation; HICs, High income countries; JBI, Joanna Briggs Institute Critical Appraisal tool; LMICs, low- and middle-income countries; MOOSE, The Meta-analysis of Observational studies in Epidemiology; NA, not available; NOS, Newcastle Ottawa Scale; PRISMA, The Preferred Reporting Items for Systematic Review and Meta-analysis; STROBE, Strengthening the Reporting of Observational Studies in Epidemiology

Table S9. Summary results from systematic reviews and meta-analyses

**First author, (No. of authors, countries)**	**Summary of results**
Balaj 2021[28]	**U5M**<br>**Maternal education:**<br>Each increase a year of education. RR= 0.970 (0.968, 0.972).<br>Primary education versus no education<br>RR= 0.839 (0.821, 0.842)<br><br>Secondary education versus no education. RR= 0.690 (0.674, 0.710)<br>Tertiary education versus no education. RR= 0.610 (0.591, 0.633)<br>**Paternal education**<br>Each increase a year of education. RR= 0.970 (0.968, 0.972).<br>Primary education versus no education<br>RR= 0.839 (0.821, 0.842)
Bhusal 2022[29]	A total of 23 studies reported 24 factors associated with U5M.<br>**Maternal age:** Ten studies examined the association of maternal age at birth of a child and U5M. it found mixed and inconclusive finding with majority revealed higher risk among younger age (<20 years).<br>**Maternal education:** Eleven studies, ten of them reported a decreased risk of U5M in children of educated or higher educational level mothers. However, one study reported increased risk among primary educated mothers compared to non-educated (AHR=1.41, 1.04, 1.91).<br>**Marital status:** marital status was investigated in three, two of them reported an increased risk of U5M among children of single mothers (AOR/AHR= 1.16 to 2.26). On study reported an increased risk of U5M among children of formerly married mothers (AOR= 1.45, 95% CI: 1.45, 2.65).<br>**Rural residence:** Eight studies examined the risk of U5M among rural residents. Five studies found significant increased risk of U5M (AOR/AHR =1.09 to 3.61). One study conducted in SSA countries showed an increased risk of U5M in four of five countries (HR=1.14-1.29, p-value<0.05), while the there was no sufficient evidence of association between rural residence and U5M in Zimbabwe. However, two studies reported a decreased risk of U5M among children in rural area (AHR/AHR= 0.79, p-value <0.05).<br>**Maternal employment status:** Five studies examined the association of maternal employment status and U5M and showed mixed finding with majority revealed an increased risk of U5M among employed or working mothers.<br>**Wealth status:** Wealth status was examined in four studies, three of them reported an increased risk of U5M among poor or lower wealth status (AHR/AOR= 1.30 to 1.43). One study low vs. Middle (HR = 1.35, CI

	= 1.08, 1.69), low vs. highest (HR = 1.30, CI = 1.02, 1.72). One study middle vs, rich (1.43, p value = 0.001), poor vs. rich (1.43, p value = 0.001). One study conducted in SSA countries showed an increased risk of U5M among poor (ref-rich) (HR= 1.01 to 1.43, p-value <0.05)<br>**Religion**: Three studies examined the association of religion and U5M showed a decreased risk among Catholic and Orthodox Christians as compared to Muslims, while one study reported an increased risk of U5M among non-religious. One study neutral/other vs. Muslim (HR = 1:43, p value = 0.02). One study orthodox vs. Muslim (IRR=0.80, CI:0.72, 0.88). One study Catholic vs. Muslim (HR = 0.84, CI = 0.73, 0.98)<br>**Ethnicity:** The association of ethnicity and U5M was examined in three studies. One study; Madhesi vs. Brahmin/Chhetri in Nepal (AHR = 1:73, CI = 1.29, 2.32, *p* value < 0.001). One study ethnic group; Peulh vs. Bwaba in rural Burkina Faso (HR = 1:39, *p* value = 0.03). One study assessed ethnic group and U5M in Nigeria found in sufficient evidence of association.<br>**Region.** The role of region was examined in five studies, showed a significant association with U5M.<br>**Family size:** Four studies reported the association of family size and U5M and showed mixed and inconclusive finding. One study; compared to ≥5, two or less (AOR = 0.37, CI = 0.22, 0.37), 3-4 (AOR = 0.38, CI = 0.31, 0.45). One study; compared to the family size of less than 6, 7-10 (HR = 0.79, CI = 0.69, 0.91), >11 (HR = 0.66, CI = 0.58, 0.76), one study: family size >5 vs. 1-5 (OR = 3.54, CI = 3.07, 4.08)<br>**Maternal age at first birth:** Three studies examined the association between age at first birth and U5M. Two studies showed an increased risk of U5M among children of mothers who gave birth before 18 years old (HR/OR 1.09 to 1.26). One study under-17 vs. ≥17 (IRR = 1.41, CI: 1.33 to 1.48).<br>**Antenatal care utilisation:** Women who utilised antenatal care had reduced risk of U5M. Three studies yes vs. no (AOR= 0.31 to 0.61). One study 1-3 vs. no (IRR = 0.841, CI = 0.737, 0.960), 4 or above vs. No (IRR = 0.814, CI = 0.702, 0.944).<br>**Contraceptive use:** The association of contraceptive use was examined in four studies found a decreased risk of U5M among mothers of contraceptive users (AHR/AOR/IRR=0.33 to 0.88). One study use of traditional contraceptive method vs. no method (OR = 0.69, CI = 0.51, 0.85). (IRR = 0.814, CI = 0.702, 0.944).<br>**Short interpregnancy interval:** Six studies examined the association of interpregnancy interval and U5M found an increased risk of U5M among shorter interpregnancy intervals (<18 months or <24 months).<br>**Multiple pregnancies**: Comparted to singleton, multiple births showed a positive significant association in seven studies included in the review (AHR/AOR=1.14 to 3.75)<br>**Caesarean section delivery:** Three studies reported increased risk of U5M after caesarean section delivery AOR/AHR= 1.20 to 1.79).<br>**Home delivery:** The association of home delivery was examined in two studies found mixed and non-conclusive finding. Howe delivery vs health facility delivery without caesarean (AOR = 0.58, CI = 0:41, 0.82, p value = 0.002). Another study home delivery vs. health facility delivery (AOR = 1.13, CI = 1.01, 1.27)<br>**History of child loss:** Two studies showed an increased risk of U5M in children of mothers with history of child death. One study found an AOR of 6.00 (p value <0.001), while the other reported an AHR of 15.97 (CI=11.64-21.92).<br>**Child gender:** Seven studies assessed child gender and U5M and showed an increased risk of U5M among male children (AHR/AOR= 1.11 to 1.61).<br>**Breastfeeding status:** Four studies investigated the association of breastfeeding status and U5M. One study; ever vs. never breastfeed (OR = 0.35, CI: 0.23, 0.51, *p* value < 0.001). One study breastfeeding duration: 13-18 vs. 0-12 months (OR = 0:14, CI = 0:072, 0:27, *p* value < 0.001), ≥19 months vs. 0-12 months (OR = 0:02, CI = 0:0064, 0:0684, *p* value < 0.001). One study; >12 months vs. <6 months (HR = 0.13, CI: 0.02, 0:84). One study: >18 vs. <6 months (OR = 0.43, CI: 0.35, 0.53).<br>**Birth order:** Six studies examined the association between birth order and U5M revealed mixed finding. One study; Second vs. first (OR = 0.41, p value = 0.009), third vs first (OR = 0.44, p value = 0.026). One study; second or third vs. first (HR = 2.60, p value < 0.05), forth or higher (HR

	= 3.77, p value < 0.05). One study: 2nd -3rd vs. first (IRR = 172, CI = 1.262, 1.491), 4th or higher vs. first (IRR = 1.487, CI = 1.373, 1.612). One study 2nd to 4th vs. first order (OR = 1.93, CI = 1.56, 2.37).<br>**Low birth weight:** three studies examined the association of low birth weight and U5M and revealed an increased in two studies (AOR=1.28 to 1:31). However, one study showed decreased risk (HR = 0.64, CI = 0.51, 0.78)<br>**Type of toilet:** Two studies examined the association of type of toilet and U5M. One study found higher odds of U5M among families with no toilet facility compared to families having flush or pit latrine (AOR = 1.037, 1.005, 1.157). Other study found higher found an increased odds of U5M among families with poor condition of latrine (OR = 1.77, CI:1.46, 2.14).<br>**Source of energy for cooking:** Four studies examined the association of source of energy for cooking and showed a reduced risk associated with use of electricity for cooking. One study electricity/gas vs. straw/animal dung (AOR = 0.920, CI: 0.811, 0.986). One study revealed that use of liquefied petroleum gas decreased risk of U5M. One study kerosene vs. gas (OR = 0.52, CI = 0.44, 0.63). One study electricity vs other sources; (OR = 0.39, CI = 0.21, 0.76).
Chikhungu 2017[30]	**Under-five mortality:**<br>Maternal death:<br>Four cohort studies including 152,201 under-five children, OR=4.09 (2.40, 6.98), I^2 = 83%, p-value =0.006. After sensitivity analysis, excluding one study, the odds ratio (OR) was 3.16 (2.27-4.38), with an I^2 of 26%.
Forde 2017[31]	**Under-five mortality:** 26 studies were included, 15 were included for meta-analysis<br>Rural place of residence; 15 studies, RR 1.47 (1.27–1.67).
Garoma 2011[32]	**Under-five mortality:**<br>Lifetime exposure to intimate partner violence; 11 studies (3 cohort, 1 case-control, 7 cross-sectional) including 87, 394 women, OR 1.52 (1.19 1.96).
Huang 2017[33]	**Under-five mortality:**<br><br>Ethnicity: 2 cross-sectional studies including 10,075, COR 2.02 (1.23, 3.32) ethnic minorities vs Han, I2=71%, P=0.001
Karimi 2020[34]	**Under-five mortality:**<br>PM2.5, PM10, carbon monoxide, sulphur dioxide and nitrogen dioxide were positively associated with infant and child mortality. However, exposure to ozone showed no association.<br><br>PM2.5 (10 µg/m^3): 6 studies (2 cohort, 2 cross-sectional, 1 case-control, 1 case-crossover), RR= 1.03 (1.02-1.05).<br><br>PM_{10} (10 µg/m3): 21 studies (3 cohort, 3 case-control, 3 cross-sectional, 7 case-crossover, 5 time-series), RR=1.03 (1.02–1.43).<br><br>CO (1 ppm): 12 studies (1 cohort, 3 case-control, 1 cross-sectional, 4 case crossover, 3 time-series). RR=1.031 (1.019-1.039).<br><br>SO_2 (1 ppm): 14 studies (2 cohort, 1 case-control, 2 cross-sectional, 4 case crossover, 3 time-series, 2 ecological), RR=1.02 (1.00–1.05).<br><br>NO_2 (1 ppm): 10 studies (2 case-control, 1 cross-sectional, 4 case-crossover, 2 time-series), RR=1.017 (1.09–1.048). These associations persisted when RR was stratified by age (infant or child) and study design.

	O_3 (1-ppb) 13 studies (1 cohort, 1 case-control, 2 cross-sectional, 6 case crossover, 3 time-series), RR= 0.989 (0.974, 1.003) I2 = 0.54%, Q = 28.2, P = 0.08).
Pretorius 2020 [35]	**Under-five mortality:**<br>Exclusive breast feeding; 6 studies (4 cohort, 1 case-control, 1 cross-sectional), involving 30,801 participants, RR=0.99 (0.97, 1.00), I^2=96.3%
Islam 2022 [8]	**Under-five mortality:**<br>Short interpregnancy interval (<24 months):<br>9 studies (1 case-control, 8 cross-sectional studies) (159, 548, 2 studies not reported)), OR 1.95 (1.56, 2.44), I2 90.3%, Egger's test (p: <0.01), 2 studies were missing, Trim and Fill estimates: OR 1.88 (1.52, 2.34)<br><br>**Infant mortality:**<br>Short interpregnancy interval (<24 months):<br>12 studies (1 case-control, 11 cross-sectional studies), (>485,773, 1 study not reported) OR 1.92 (1.77, 2.07), I2 51.4%, Egger's test (p: <0.01), 2 studies were missing, Trim and Fill estimates: OR 1.88 (1.52, 2.34). Under-five mortality: 9 studies (1 case-control, 8 cross-sectional studies) (159, 548, 2 studies not reported)), OR 1.95 (1.56, 2.44), I2 90.3%, Egger's test (p: <0.01), 2 studies were missing, Trim and Fill estimates: OR 1.88 (1.52, 2.34)
Karami 2024 [36]	**Under-five mortality:**<br>The review reported significant association between socioeconomic status (4 studies), birth weight (3 studies) and mode of delivery (2 studies) with U5M without indicating the direction of association. Mixed finding was reported for maternal education (6 studies), while non-significant association was found for paternal education (2 studies), maternal age (2 studies), residence (3 studies), and maternal smoking (2 studies).<br><br>**Infant mortality:**<br>A review found significant association for GDP per capita (2 studies), birth weight (5 studies), residence (3 studies), preterm birth (2 studies), history of still birth (3 studies), history of abortion (2) with Infant mortality. The direction of associations was not indicated. There was mixed finding for maternal education (7 studies), paternal education (5 studies), socioeconomic status (7 studies), mode of delivery (5 studies), birth interval (6 studies), and infant gender (5 studies). Non-significant associations were reported for maternal age (3 studies), gravidity (2), and birth order (2 studies).
Adane 2021[37]	**Infant mortality**<br>**Maternal anxiety and depression:** Five studies and 477,127 births, OR 1.47 (0.92, 2.35): I^2=63.3%.<br>**Severe mental illness:** seven studies included 6,812,821 births, OR, 1.42 (1.08–1.87), I^2=56.3%.<br>**Any maternal mental illness:** 11 studies involving 6,861,091 births 1.72 (1.40-2.11), I2=83.3%.<br>No evidence of publication bias.
Ahrens 2018[38]	Short interpregnancy interval and infant mortality was examined in four studies involving a total of 220, 676 participants. Three studies showed an increased risk of infant mortality among short interpregnancy intervals (<6 months), with AOR ranging from 1.44 (95% CI: 1.06, 1.95) to

	2.23 (95% CI: 1.19, 4.16), compared to the reference interpregnancy interval 18-23 months. One study showed an increased risk of infant mortality interpregnancy intervals of 6-11 months (aOR=1.68), and 12-17 months (aOR=1.48).
Aune 2014[39]	**Infant mortality:**<br>Body mass index increase per 5units; 4 studies, 4983 deaths 1,491,879 births, RR=1.18 (1.09-1.28), I^2 = 79.0%; P = .003.<br>No evidence of publication bias using the Egger test (P = 0.56).<br>In sensitivity analyses that excluded one study at a time from each analysis, the result appeared to be robust to the influence of individual studies.
Bagade 2018[40]	Two studies examining the association of physical, sexual, and emotional violence with infant mortality found an increased risk of infant mortality.
Brennan 2016[41]	**Infant mortality**:<br>Exposure to HIV, 22 cohort studies, and 29212 infants, RR= 1.70 (1.30, 2.22), I^2=73.9.<br>No publication bias.
Brocklehurst 1998[42]	**Infant mo**rtality:<br>Maternal HIV infection (Compared to children of HIV uninfected mother):<br>Nine studies including 1876 HIV-infected and 4960 HIV-uninfected women. OR=3.69 (3.03, 4.49), X^2=30.7, P < 0.001. Publication bias not reported.
Dadi 2015[43]	Infant mortality: Short interpregnancy interval; 5 studies involving 43,909 infants, OR 2.03 (1.52-2.70) I2=70%, P<0.05
Eltayib 2023[44]	**Infant mortality:**<br>Female newborn RR of 4 studies: 1.473 (1.129–1.921) I2 = 0.01%, Q = 1.594, p = 0.661) The Egger's test p = 0.9024, and Begg's test p = 0.497.<br>Consanguineous marriage, RR of 2 studies: 1.507 (1.182–1.920) I2 = 0.001%, Q = 0.009, p = 0.9227) (Egger's test p = 0.435, Begg's test p = 0.317).<br>Paternal education (Primary and below educated vs secondary or above), RR of 2 studies: 1.56 (1.102–2.207 (I2 = 0.01%, Q = 0.075, p = 0.785) with Begg's test p = 0.431, Egger's test p = 0.3173.<br>Maternal education (Primary and below educated vs secondary or above), RR of 2 studies: 2.018 (95% CI = 1.486–2.741) (I2 = 0.01%, Q = 0.018 623 p = 0.895) with Begg's test p = 0.0976, Egger's test p= 0.3173.<br>Paternal occupation (unemployed vs employed), RR of 2 studies: 2.315 (1.399–3.829) (I2 = 0.01%, Q = 0.225, p = 0.635) with Begg's test p = 0.793, Egger's test p = 0.3173.<br>Maternal occupation (housewife vs working), RR of 2 studies: 1.117 (95% CI = 0.883–1.413) (I2 = 0.01%, Q = 0.009, p = 0.923) with Begg's test p = 0.215, Egger's test p = 0.3173.<br>Low birth weight (LBW) infants (<2500 g) RR of 4 studies: 11.578 (95% CI = 3.202–41.870); (I2 = 91.18%, Q = 22.67, p = 0.097) Egger's test p = 0.5981, and Begg's test p = 0.602.<br>Gestational age (preterm) 6 studies RR: 10.679 (95% CI = 5.595–20.384), (I2 = 90.63%, Q = 53.34, p = 0.078). (Egger's test, p = 0.274, Begg's test, p = 0.191).<br>Foetal growth (Small for gestational age), RR of 2 studies 6.825 (95% CI = 4.636–10.046), (I2 = 0.01%, Q = 0.822, p = 0.365) with a Begg's test p = 0.198, and Egger's test p = 0.317.<br>Fetal growth (Appropriate for gestational age vs Large for gestational age)2 studies became 6.413 (95% CI = 2.061–19.950). (I2 = 0.01%, Q = 0.047, p = 0.828) with a Begg's test p = 0.154, and Egger's test p = 0.317).

	Foetal growth (Small for gestational age vs Appropriate for gestational age) 3 studies 13.008 (95% CI = 6.525–25.933). (I2 = 24.08%, Q = 2.635, p = 0.268) with a Begg's p = 0.717, and Egger's p = 0.602.<br>APGAR score at 1 min (7 or above vs less than 7) RR of the 2 studies became 7.465 (95% CI = 4.593–12.134), (I2 = 0.01%, Q = 0.003, p = 0.959) with a Begg's test p = 0.301, and Egger's test p = 0.317.<br>APGAR score at 5 min (7 or above vs less than 7) RR of 2 studies became 3.998 (95% CI = 2.902-5.508) (I2 = 43.05%, Q = 1.756, p = 0.185) with a Begg's test p = 0.315 and Egger's test p = 0.317.<br>Mode of delivery (vaginal vs caesarean section) RR of 3 studies 2.295 (95% CI = 1.476–3.566), (I2 = 60.68%, Q = 2.543, p = 0.111) with Egger's test p = 0.111 and Begg's test p = 0.31.<br>ANC (No vs yes) RR based on 2 studies was 3.241 (95% CI = 2.527–4.156) (I2 = 001%, Q = 0.186, p = 0.666) with Egger's test p = 0.813, and Begg's test p = 0.317).<br>Multiple pregnancy vs singleton pregnancy RR based on 2 studies 7.585 (95% CI = 5.208–11.045) (I2 = 44.93%, Q = 2.219, p = 0.136) Egger's test p = 0.132, Begg's test p = 0.3173.<br>Gravidity (4 or above vs 3 or fewer) RR of two studies 1.036 (95% CI = 0.852–1.261). (I2 = 0.001%, Q = 0.282, p = 0.595). The Egger's test p = 0.149, and Begg's test, p = 0.317.<br>Foetal presentation (breech vs cephalic) RR of 2 studies 3.974 (95% CI = 1.697–9.307). (I2 = 83.10%, Q = 5.919, p = 0.055) with Begg's test p = 0.3173, Egger's test p = 0.176.
Glinianaia 2004[45]	Eight studies examined particulate air pollution and infant mortality found little evidence of a consistent association. Five studies reported positive associations, although the strength of evidence and critical exposure period differed. Three other studies reported nonsignificant associations.
Huo 2021[46]	The review reported significant association between socioeconomic status (4 studies), birth weight (3 studies) and mode of delivery (2 studies) with U5M without indicating the direction of association. Mixed finding was reported for maternal education (6 studies), while non-significant association was found for paternal education (2 studies), maternal age (2 studies), residence (3 studies), and maternal smoking (2 studies).<br>A review found significant association for GDP per capita (2 studies), birth weight (5 studies), residence (3 studies), preterm birth (2 studies), history of still birth (3 studies), history of abortion (2) with Infant mortality. The direction of associations was not indicated. There was mixed finding for maternal education (7 studies), paternal education (5 studies), socioeconomic status (7 studies), mode of delivery (5 studies), birth interval (6 studies), and infant gender (5 studies). Non-significant associations were reported for maternal age (3 studies), gravidity (2), and birth order (2 studies).
Jacques 2019[47]	**Infant mortality**:<br>Postnatal depressive symptoms or depression: 3 cohort studies, including 140,275 participants RR 1.93 (1.02, 1.64), asymmetrical funnel plot, I^2= 92.0%
Jahan 2007[48]	Infant Mortality: Poor<br>9 studies, OR =1.52, (1.38, 1.67)
Kiross 2019[49]	**Infant mortality:**<br>Primary education; 5 studies involving 29557 participants, OR 0.72 (0.66-0.78), no evidence of publication bias using the funnel plot, I2=0% (p = 0.63).<br>Secondary and above education; 5 studies involving 26582 OR=0.55 (0.47-0.64), no evidence of publication bias using the funnel plot, I^2=17% (p = 0. 30)

Kozuki 2013[50]	**Infant mortality:** The review reported significant association between short birth interval (<18 months) and infant mortality compared to 36-<60 months birth interval. <18 months, aOR 1.83 (1.19, 2.81). 18-<24 months, aOR 1.08 (0.66, 1.78). 24-<36 months; aOR 1.17 (0.96, 1.43). ≥60 months, aOR 1.01 (0.84, 1.22).
Luben 2023[51]	**Infant mortality:** PM_{10}-Infant mortality; 16 studies reported the association between short-term exposure to PM_{10} and infant mortality, 14 (7 case-crossover, 4 time-series, 2 case-control and 1 cross-sectional) were included for the meta-analysis. The random effect pooled estimate for PM_{10}, OR 1.02 (1.00, 1.03). I^2=66 % and Q statistic p-value (<0.0005). Egger's test (p = 0.40), trim and fill analyses estimated no missing studies. O_3 – infant mortality; 13 of the 15 studies (6 case-crossover,7 time-series, 2 case-control and 1 cross-sectional) were included for the meta-analysis. The random effect pooled estimate for O_3, OR 0.99 (0.97, 1.01). I2=93 % and Q statistic p-value (<0.001). Egger's test (p = 0.40), trim and fill analyses estimated no missing studies. leave-one-out sensitivity analyses showed no substantial influence on the pooled estimate. NO_2 – infant mortality; 11 (7 time-series and 4 case-crossover) of 14 studies included for meta-analysis. The random effect pooled estimate, OR 1.04 (1.01-1.08). I2=87 % and Q statistic p-value (<0.002). Egger's test (p = 0.001), trim and fill analyses estimated 5 missing studies. leave-one-out sensitivity analyses showed some influence on the pooled estimate. The trim and fill analyses estimated five missing studies and resulted in a pooled odds ratio of 1.01 (1.00, 1.01) SO_2 – infant mortality; 8 of 11 studies (4 time-series and 4 case-crossover) were included for the meta-analysis. The random effect pooled estimate, OR 1.07 (1.02, 1.12). I2=61% and Q statistic p-value (<0.005). Egger's test (p = 0.37), trim and fill analyses estimated one missing study and resulted in a pooled odds ratio of 1.07 (1.02, 1.12). Leave-one-out sensitivity analyses showed no substantial influence on the pooled estimate. CO – infant mortality; 8 of 11 studies were included for the meta-analysis. The random effect pooled estimate, OR 1.01 (1.00, 1.02). I2=54% and Q statistic p-value (<0.0002). Egger's test (p <0.001), trim and fill analyses estimated 5 missing studies. leave-one-out sensitivity analyses showed some influence on the pooled estimate. The trim and fill analyses estimated five missing studies and resulted in a pooled odds ratio of 1.01 (1.00, 1.01).
Mazzone 2023[52]	**Infant mortality:**<br>Maternal epilepsy; 13 studies involving 1,426,692 reported, OR 1.87 (1.56-2.24), I^2=31%
Meehan 2014[53]	**Infant mortality:**<br>For all obese mothers with a BMI greater than or equals to 30 the odds of having an infant death, 11 studies including 3,230,917 participants, OR 1.42 (1.24, 1.63), P<0.001, I2 = 56%.<br>The exposure of overweight or obese mothers (BMI >25) compared with normal BMI was significantly associated with greater odds of infant death, 11 studies including 3,230,917 participants, OR 1.27 (1.14, 1.42), P<0.001, I2 =63%.<br>There was also an increasing trend in the pooled OR of infant death with increased maternal BMI, with BMI greater than or equals to 35 having the highest, 3 studies involving 1,423,013, OR 2.03 (1.61, 2.56), P< 0.001, I2 = 0%; 3 studies. Sensitivity analysis showed similar pooled effect measures. No publication bias using funnel plot.
Nguyen 2019[54]	**Infant mortality:**<br>Maternal death: For children whose mother died when they were ≤ 0–12 months, the RR 15.9 (2.2-116.1), I2=82.9%, p= 0.02, pooled estimate from 2 studies and 37,853 participants.
Quansah 2015[55]	**Infant mortality.**<br>**Arsenic exposure:** 7 studies OR: 1.35 (1.12, 1.62) (I2 = 30.4%, p = 0.196). A funnel plot showed evidence of asymmetry, suggesting influence of small positive studies. The strength of association was attenuated with the trim and fill method, and three missing studies were imputed.
Rahman 2021[56]	**Infant mortality:**<br>Infant resided in rural (compared to urban), OR 1.51 (1.42-1.60), there was evidence of publication bias

Sankar 2015[57]	**Infant mortality:**<br>Compared to exclusively breastfed infants, predominantly breastfed infants aged 0–5 months, 3 studies (22, 048 Participants), RR 0.67 (0.52, 0.88), I2=0%.<br>Exclusive vs partially breastfed infants, 3 studies (22, 048 Participants), RR 0.35 (0.20, 0.61), I2=68.3%.<br>Exclusive vs not breastfed: 2 studies (11, 101), RR 0.07 (0.03, 0.16). I2=70.5%
Weightman 2012[58]	**Infant mortality:**<br>Area deprivation (highest vs lowest area deprivation quintiles); 3 studies OR 1,72(1.37-2.15), heterogeneity p=0.002. Infant mortality: social class (lowest vs highest social class); 3 studies, OR 1.52 (1.44-1.61), heterogeneity p=0.928.

Table S10. Summary of evidence of meta-analyses on under-five mortality.

Risk factor	Meta-analysis	No of primary studies; study design	Total participants	Evidence of significant publication bias	I^2 (%)	OR (95% CI)	Consistency, Confidence
**Maternal education (Ref-not educated)**							
Each increase a year of education	Balaj 2021[28]	300; Incomplete	>3,112,474	NA	NA	0.970 (0.968, 0.972)	NG
Primary education	Balaj 2021[28]	300; Incomplete	>3,112,474	NA	NA	0.839 (0.821, 0.842)	NG
Secondary education	Balaj 2021[28]	300; Incomplete	>3,112,474	NA	NA	0.690 (0.674, 0.710)	NG
Tertiary education	Balaj 2021[28]	300; Incomplete	>3,112,474	NA	NA	0.610 (0.591, 0.633)	NG
**Paternal education**							
Each additional year of education	Balaj 2021[28]	NA	>3,112,474	NA	NA	0.984 (0.983, 0.987)	NG
Primary education	Balaj 2021[28]	NA	>3,112,474	NA	NA	0.909 (0.901, 0.922)	NG
Secondary education	Balaj 2021[28]	NA	>3,112,474	NA	NA	0.827 (0.812, 0.850)	NG
Tertiary education	Balaj 2021[28]	NA	>3,112,474	NA	NA	0.776 (0.758, 0.805)	NG
**Residence** (rural vs urban)	Forde 2017[31]	15; all cross-sectional	NA	No	0	1.47 (1.27, 1.67)	++, Ls
**Ethnicity** (minorities vs Han in China)	Huang 2017[33]	2; all cross-sectional	10,075	NA	71	1.99 (1.23, 3.32)	?, Lnc
**Short birth interval** (< 24 months) (yes vs no)	Islam 2022 [8]	9; 1 case-control, 8 cross-sectional	>159, 548	Yes	90.3	1.95 (1.56, 2.44)	++, Ls

**Intimate partner violence against women**	Garoma 2011[32]	11; 3 cohort, 1 case-control, 7 cross-sectional	87, 394	No	NA	1.52 (1.19, 1.96)	+, Ls
**Breastfeeding status**							
Exclusive vs. non-exclusive breastfed	Pretorius 2020[35]	6; 4 cohort, 1 case-control, 1 cross-sectional	30,801	No	96.3	0.18 (0.06, 0.46)	++, Pe
**Maternal death**	Chikhungu 2017[30]	4; all cohort	152,201	No	83	4.09 (2.40, 6.98)	++, Pe
**Air pollution**							
$PM_{2.5}$ (10 µg/m^3)	Karimi 2020[34]	6; 2 cohort, 2 cross-sectional, 1 case-control, 1 case-crossover	NA	NA	81.2	1.03 (1.02, 1.05)	?, Ls
PM_{10} (10 µg/m^3)	Karimi 2020[34]	21; 3 cohort, 3 case-control, 3 cross-sectional, 7 case-crossover, 5 time-series,	NA	NA	80.8	1.03 (1.02, 1.43)	?, Ls
NO_2 (1 ppb)	Karimi 2020[34]	10; 2 case-control, 1 cross-sectional, 4 case-crossover, 2 time-series	NA	NA	67.3	1.11 (1.05, 1.30)	?, Ls
SO_2 (1 ppb)	Karimi 2020[34]	14; 2 cohort, 1 case-control, 2 cross-sectional, 4 case crossover, 3 time-series, 2 ecological	NA	NA	46.2	1.08 (1.04, 1.12)	?, Ls
O_3 (1 ppb)	Karimi 2020[34]	13; 1 cohort, 1 case-control, 2 cross-sectional, 6 case crossover, 3 time-series,	NA	NA	54	0.95 (0.86, 1.00)	?, Ls
CO (1 ppm)	Karimi 2020[34]	12; 1 cohort, 3 case-control, 1 cross-sectional, 4 case crossover, 3 time-series,	NA	NA	0	1.03 (1.02, 1.04)	?, Ls

Note: NA, not available; NG, Not graded; $PM_{2.5}$, particulate matters with aerodynamic diameter ≤2.5µm; PM_{10}, particulate matters with aerodynamic diameter ≤10µm; CO, carbon monoxide; SO_2, sulphur dioxide; NO_2, nitrogen dioxide; '++' indicates consistent positive association; '+' indicates less consistent positive association '?' indicates unclear or contradictory direction; Ls, limited suggestive evidence; Pe, probable evidence; Lnc, limited no conclusive evidence

Table S11. Summary of evidence of meta-analyses on infant mortality

Risk factors	Meta-analysis	No of primary studies; study design	Total participants	Evidence of significant publication bias	I^2 (%)	RR (95% CI)	Consistency, confidence
**Socio-demographic and economic factors**							
Maternal education (secondary or above vs. primary or below)	Eltayib 2023[44]	2; 1 cohort, 1 cross-sectional	56,022	No	0.01	0.45 (0.36, 0.67)	--, Lnc
Maternal education (primary vs. not educated)	Kiross 2019[49]	5; 1 cohort, 1 case-control, 3 cross-sectional	29,557	No	0	0.74 (0.68, 0.80)	--, Ls
Maternal education (Secondary vs. not educated)	Kiross 2019[49]	5; 1 cohort, 1 case-control, 3 cross-sectional	26,582	No	17	0.58 (0.50, 0.67)	--, Ls
Paternal education (Secondary or above vs. primary or below)	Eltayib 2023[44]	2; 1 cohort, 1 cross-sectional	55,687	No	0.01	0.64 (0.45, 0.91)	--, Lnc
Maternal employment (housewife vs. employed)	Eltayib 2023[44]	2; 1 cohort, 1 cross-sectional	62,555	No	0.01	1.12 (0.88, 1.41)	00, Lnc
Paternal employment (unemployed vs. employed)	Eltayib 2023[44]	2; 1 cohort, 1 cross-sectional	55,491	No	0.01	2.32 (1.40, 3.83)	++, Lnc
Consanguineous marriage	Eltayib 2023[44]	2; 1 cohort, 1 cross-sectional	55,432	No	0.001	1.51 (1.18, 1.92)	++, Lnc
Rural residence	Rahman 2021[56]	65; all cross-sectional	NA	No	NA	1.51 (1.42, 1.60)	+, Ls
Poor household	Jahan 2007[48]	9; 2 case-control, 7 cross-sectional	NA	NA	NA	1.52. (1.38, 1.67)	++, Ls
Lowest social class	Weightman 2012[58]	3; all cohort	NA	NA	Yes	1.52 (1.44, 1.61)	+, Ls
Area deprivation (highest vs lowest quintile)	Weightman 2012[58]	3; all cohort	NA	NA	Yes	1,72(1.37, 2.15)	+, Ls
**Maternal nutrition and reproductive health-related factors**							
Short birth interval <24 months (Yes vs. No)	Dadi 2015[43]	5; 1 cohort, 2 case-control, 2 cross-sectional	43,909	Yes	70	1.89 (1.47, 2.41)	++, Ls

	Islam 2022 [8]	12; 1 case-control, 11 cross-sectional	>485,773	Yes	51.4	1.92 (1.77, 2.07)	
Interpregnancy interval (Ref_36-60 months)							
Less than 18 months	Kozuki 2013[50]	5; all cohort	19,240	NA	NA	1.83 (1.19, 2.81)	+, Pe
18 - 24 months	Kozuki 2013[50]	5; all cohort	19,240	NA	NA	1.08 (0.66, 1.78)	?, Ls
24 - 36 months	Kozuki 2013[50]	5; all cohort	19,240	NA	NA	1.17 (0.96, 1.43)	?, Ls
≥60 months	Kozuki 2013[50]	5; all cohort	19,240	NA	NA	1.01 (0.84, 1.22)	00, Pe
Multiple pregnancy	Eltayib 2023[44]	2; all cross-sectional	62,898	NA	44.93	7.59 (5.21, 11.04)	++, Lnc
Gravidity (4 or more vs. 3 or fewer)	Eltayib 2023[44]	2; 1 cohort, 1 cross-sectional	73,360	No	0.001	1.04 (0.85, 1.26)	00, Ls
Mode of delivery (Vaginal vs. CS)	Eltayib 2023[44]	2; 1 cohort, 1 cross-sectional	312	No	60.68	2.29 (1.48, 3.57)	++, Lnc
Antenatal care utilisation	Eltayib 2023[44]	2; 1 cohort, 1 cross-sectional	72,985	No	0.01	0.31 (0.24, 0.40)	--, Lnc
**Maternal nutrition**							
Underweight vs. healthy weight	Huo 2021[46]	7; all cohort	10,044,959	No	0	0.93 (0.88, 0.98)	?, Ls
Overweight vs. healthy weight	Huo 2021[46]	8; all cohort	10,762,039	No	0	1.16 (1.13, 1.19)	?, Ls
Overweight or obese vs. healthy weight	Meehan 2014[53]	11; 10 cohort, 1 case-control	3,230,917	No	63	1.27 (1.14, 1.42)	NG
Obesity vs. normal weight	Huo 2021[46]	7; all cohort	10,762,039	No	88.7	1.55 (1.41, 1.70)	++, Pe
	Meehan 2014[53]	11; 10 cohort, 1 case-control	3,230,917	No	56	1.42 (1.24, 1.63)	
BMI ≥35kg/m^2 vs. healthy weight	Meehan 2014[53]	3; NA	1,423,013	No	0	2.03 (1.61, 2.56)	NG
BMI (5-unit increase)	Aune 2014[39]	4; all cohort	1,491,879	No	79.0	1.18 (1.09, 1.28)	++, Pe
**Infant-related factors**							
Infant gender (Female vs. male)	Eltayib 2023[44]	4; 3 cohort, 1 cross-sectional	465	No	0.01	1.47 (1.13, 1.92)	+, Ls
Preterm birth (<37 weeks)	Eltayib 2023[44]	6; 2 cohort, 4 cross-sectional	84,695	No	90.63	10.68 (5.60, 20.38)	++, Ls

Low birth weight (<2500 gm)	Eltayib 2023[44]	3; 2 2222cohort, 1 cross-sectional	15,080	No	91.18	11.58 (3.20, 41.87)	++, Ls
SGA vs. AGA	Eltayib 2023[44]	2; 1 cohort, 1 cross-sectional	25,145	No	0.01	6.83 (4.64, 10.05)	++, Lnc
AGA vs. LGA	Eltayib 2023[44]	2; all cohort	14,588	No	0.01	6.41 (2.06, 19.95)	++, Ls
SGA vs. LGA	Eltayib 2023[44]	3; 2 cohort, 1 cross-sectional	5,522	No	24.08	13.01 (6.52, 25.93)	++, Ls
Low APGAR score at 1 min	Eltayib 2023[44]	2; 1 cohort, 1 cross-sectional	246	No	0.01	7.46 (4.59, 12.13)	++, Lnc
Low APGAR score at 5 min	Eltayib 2023[44]	2; 1 cohort, 1 cross-sectional	246	No	43.05	4.00 (2.90, 5.51)	++, Lnc
Breach foetal presentation	Eltayib 2023[44]	2; all cohort	14,919	No	83.10	3.97 (1.70, 9.31)	++, Ls
Exclusive vs. predominant breastfed	Sankar 2015[57]	3; 1 cohort, 2 secondary analysis of RCT	22,048	NA	0	0.67 (0.52, 0.88)	?, Lnc
Exclusive vs, partial breastfed	Sankar 2015[57]	3; 1 cohort, 2 secondary analysis of RCT	22.048	NA	68.3	0.35 (0.20, 0.61)	--, Lnc
Exclusive vs Not breastfed	Sankar 2015[57]	2; 1 cohort, 1 secondary analysis of RCT	11,101	NA	70.5	0.07 (0.03, 0.16)	--, Lnc
Exposed to HIV (unexposed)	Brennan 2016[41]	22; all cohort	29,212	No	73.9	1.70 (1.30, 2.22)	?, Ls
**Maternal morbidity and mortality**							
Maternal anxiety and depression	Adane 2021[37]	5; NA	477,127	No	63.3	1.47 (0.92, 2.35)	NG
Postnatal depressive symptoms or depression	Jacques 2019[47]	3; all cohort	140,275	No	92.0	1.93 (1.02, 1.64)	+, Ls
Severe mental illness	Adane 2021[37]	7; NA	6,812,821	No	56.3	1.42 (1.08, 1.87)	NG
Any maternal mental illness	Adane 2021[37]	11; NA	6,861,091	No	83.3	1.61 (1.17, 2.21)	NG
Maternal HIV infection	Brocklehurst 1998[42]	9; all cohort	6836	NA	NA	2.99 (2.58, 3.44)	++, Pe
Maternal epilepsy	Mazzone 2023[52]	13; NA	1,426,692	NA	31	1.87 (1.56, 2.24)	NG
Maternal death	Nguyen 2019[54]	2; 1 cohort, 1 not specified	37,853	NA	82.9	15.9 (2.2-116.1)	++, Lnc
**Environmental factors**							

PM_{10} (10 µg/m3)	Luben 2023[51]	14; 7 case-crossover, 4 time-series, 2 case-control, 1 cross-sectional	NA	No	66	1.02 (1.00, 1.03)	?, Ls
NO_2 (10 ppb)	Luben 2023[51]	11; 7 time-series, 4 case-crossover	NA	Yes	87	1.23 (1.05, 1.50)	?, Ls
SO_2 (10 ppb)	Luben 2023[51]	8; 4 time-series, 4 case-crossover	NA	No	61	1.29 (1.08, 1.54)	?, Ls
O_3 (10 ppb)	Luben 2023[51]	13; 6 case-crossover,7 time-series, 2 case-control, 1 cross-sectional	NA	No	93	0.95 (0.86, 1.05)	?, Ls
CO (1 ppm)	Luben 2023[51]	11; 6 time-series, 4 case-crossover, 1 cross-sectional	NA	Yes	54	1.01 (1.00, 1.02)	?, Ls
Arsenic exposure	Quansah 2015[55]	7; 5 cohort, 2 cross-sectional	>43,616	NA	30.4	1.35 (1.12, 1.62)	?, Ls

Note: NA, not available; NG, Not graded; CO, carbon monoxide; NO_2, nitrogen dioxide; O_3, ozone; '++' indicates consistent positive association; '+' indicates less consistent positive association '?' indicates unclear or contradictory direction; '--' indicates consistent negative association; Ls, limited suggestive evidence; Pe, probable evidence; Lnc, limited no conclusive evidence

Table S12. Study overlaps in systematic reviews

Meta-analyses reported similar factors	Total number of times studies appeared in the meta-analyses (N)	Number of indexed primary study (r)	Number of reviews (c)	Corrected covered area (CCA in %)	Degree of overlap
Meta-analyses on short interpregnancy interval (<24 months)	17	17	2	0	No
Meta-analyses on maternal obesity (BMI >30 kg/m^2)	18	15	2	0.2	Slight

$$CCA = \frac{N - r}{rc - r}$$

Where N is the total number of primary studies include in the reviews, r is the total number of indexed primary studies, and c is the total number of reviews. The overlaps classified based on CCA score as slight (≤5%), moderate (6-10%), high (11-15%), and very high (>15%).[59]

**Review**	**Items**																**Overall quality of the review**
	**1**	**2**	**3**	**4**	**5**	**6**	**7**	**8**	**9**	**10**	**11**	**12**	**13**	**14**	**15**	**16**	

Adane 2021	Y	Y	N	Y	Y	Y	Y	Y	Y	N	Y	Y	Y	Y	Y	Y	Moderate
Ahrens 2018	Y	Y	Y	N	Y	Y	Y	Y	Y	N	NA	NA	Y	Y	NA	Y	Moderate
Aune 2014	N	N	Y	PY	N	N	Y	N	Y	N	Y	Y	Y	Y	Y	Y	Low
Bagade 2018	N	Y	Y	Y	Y	Y	Y	Y	Y	N	NA	NA	Y	Y	NA	Y	Low
Balaj 2021	Y	Y	Y	Y	Y	N	Y	N	Y	N	Y	Y	Y	Y	Y	Y	Moderate
Bhusal 2022	N	N	N	PY	Y	Y	Y	Y	N	N	NA	NA	N	N	NA	Y	Critically low
Brennan 2016	N	N	N	PY	Y	N	Y	PY	Y	N	Y	Y	Y	Y	Y	Y	Low
Brocklehurst 1998	N	PY	N	PY	N	N	N	PY	N	N	Y	Y	Y	Y	N	N	Low
Chikhungu 2017	Y	N	N	PY	N	N	Y	Y	Y	N	Y	N	Y	Y	Y	Y	Low
Dadi 2015	N	N	N	PY	Y	N	Y	Y	N	N	Y	N	Y	Y	Y	Y	Critically low
Eltayib 2023	N	N	N	Y	Y	Y	Y	N	Y	N	Y	Y	Y	Y	Y	Y	Low
Forde 2017	N	N	N	PY	Y	N	N	N	N	N	Y	Y	Y	Y	Y	Y	Critically low
Garoma 2011	N	N	N	N	N	N	Y	PY	N	N	Y	Y	Y	N	Y	N	Critically low
Glinianaia 2024	N	N	N	N	N	N	Y	PY	N	N	NA	NA	Y	Y	NA	Y	Critically low
Huang 2017	Y	N	N	Y	N	Y	Y	Y	Y	N	Y	Y	Y	Y	Y	Y	Low
Huo 2021	N	N	N	Y	N	Y	Y	Y	Y	N	Y	Y	Y	Y	Y	Y	Low
Islam 2022	Y	N	N	Y	Y	Y	Y	PY	Y	N	Y	Y	Y	Y	Y	Y	Low
Jacques 2019	N	N	N	PY	Y	N	Y	Y	Y	N	Y	Y	Y	Y	Y	Y	Low
Jahan 2007	N	N	N	PY	N	N	Y	Y	N	N	Y	Y	Y	Y	N	Y	Critically low
Karimi 2020	N	Y	N	Y	N	Y	Y	Y	Y	Y	Y	Y	Y	Y	Y	Y	Moderate
Karami 2024	N	N	N	PY	N	N	Y	N	Y	N	NA	NA	Y	N	NA	Y	Low
Kiross 2019	N	N	N	PY	N	Y	Y	Y	Y	N	Y	Y	Y	Y	Y	Y	Low
Kozuki 2013	N	N	N	N	N	N	N	PY	N	N	Y	Y	Y	N	N	Y	Critically low
Luben 2023	Y	N	N	PY	Y	Y	Y	PY	Y	N	Y	Y	Y	Y	Y	Y	Low
Mazzone 2023	N	Y	N	Y	Y	Y	Y	PY	Y	N	Y	Y	Y	Y	N	N	Low
Meehan 2014	N	Y	N	PY	Y	Y	Y	PY	Y	N	Y	Y	Y	Y	Y	Y	Moderate
Nguyen 2019	N	N	N	PY	N	Y	Y	Y	Y	N	Y	Y	Y	Y	Y	Y	Low
Pretorius 2020	N	N	N	PY	Y	N	Y	PY	Y	N	Y	Y	Y	Y	Y	Y	Low
Quansah 2015	N	N	N	PY	Y	Y	Y	Y	Y	N	Y	Y	Y	Y	Y	Y	Low
Sankar 2015	Y	N	Y	Y	N	N	Y	Y	Y	N	Y	Y	Y	Y	N	Y	Critically low

Rahman 2021	N	N	N	PY	N	N	Y	N	N	N	Y	Y	Y	N	Y	N	Critically low
Weightman 2012	N	N	N	Y	Y	Y	Y	PY	Y	N	Y	Y	Y	Y	Y	Y	Low

Note: Y, yes; PY, partially yes; N, No; NA, not applicable

1. Did the research questions and inclusion criteria for the review include the components of population, intervention, comparison, and outcome?
2. Did the report of the review contain an explicit statement that the review methods were established prior to the conduct of the review and did the report justify any significant deviations from the protocol?
3. Did the review authors explain their selection of the study designs for inclusion in the review?
4. Did the review authors use a comprehensive literature search strategy?
5. Did the review authors perform study selection in duplicate?
6. Did the review authors perform data extraction in duplicate?
7. Did the review authors provide a list of excluded studies and justify the exclusions?
8. Did the review authors describe the included studies in adequate detail?
9. Did the review authors use a satisfactory technique for assessing the risk of bias (RoB) in individual studies that were included in the review?
10. Did the review authors report on the sources of funding for the studies included in the review?
11. If meta-analysis was performed did the review authors use appropriate methods for statistical combination of results?
12. If meta-analysis was performed, did the review authors assess the potential impact of RoB in individual studies on the results of the meta-analysis or other evidence synthesis?
13. Did the review authors account for RoB in individual studies when interpreting/ discussing the results of the review?
14. Did the review authors provide a satisfactory explanation for, and discussion of, any heterogeneity observed in the results of the review?
15. If they performed quantitative synthesis did the review authors carry out an adequate investigation of publication bias (small study bias) and discuss its likely impact on the results of the review?
16. Did the review authors report any potential sources of conflict of interest, including any funding they received for conducting the review?

NB: Items 2, 4, 7, 9, 11, 13 and 15 are critical.

Figure S1. Quality assessment using AMSTAR 2 tool.

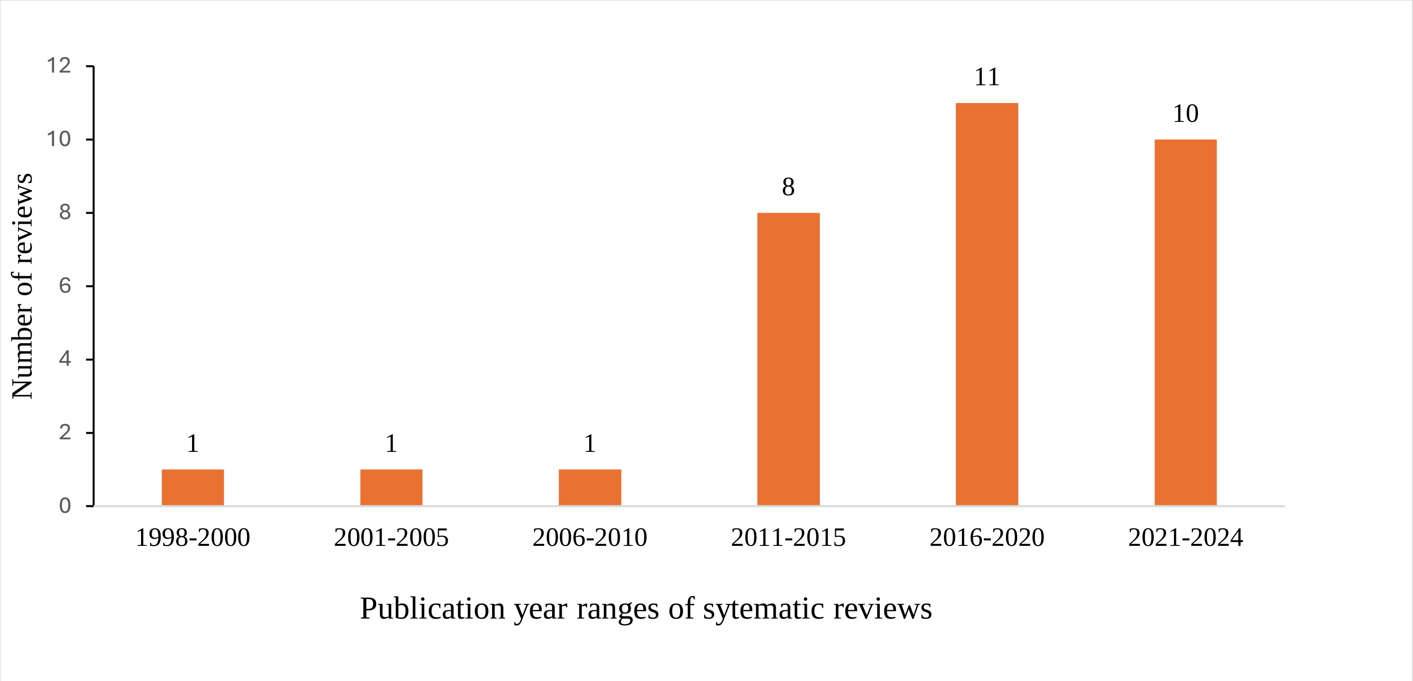

Figure S2. Publication date ranges of systematic reviews

References

1 Shrier I, Steele R. Understanding the relationship between risks and odds ratios. Clin J Sport Med. 2006;16(2):107-10.

2 Borroni E, Pesatori AC, Bollati V, et al. Air pollution exposure and depression: A comprehensive updated systematic review and meta-analysis. Environmental Pollution. 2022;292:118245.

3 Mehretie Adinew Y, Feleke SA, Mengesha ZB, et al. Childhood mortality: trends and determinants in Ethiopia from 1990 to 2015—a systematic review. Advances in Public Health. 2017;2017

4 Bede-Ojimadu O, Orisakwe OE. Exposure to Wood Smoke and Associated Health Effects in Sub-Saharan Africa: A Systematic Review. Annals of Global Health. 2020;86(1):32.

5 Doku DT, Bhutta ZA, Neupane S. Associations of women's empowerment with neonatal, infant and under-5 mortality in low- and /middle-income countries: meta-analysis of individual participant data from 59 countries. BMJ Global Health. 2020;5(1):e001558.

6 Gissler M, Alexander S, MacFarlane A, et al. Stillbirths and infant deaths among migrants in industrialized countries. Acta Obstetricia et Gynecologica Scandinavica. 2009;88(2):134-48.

7 Islam MA, Tabassum T. Does antenatal and post-natal program reduce infant mortality? A meta-analytical review on 24 developing countries based on Demographic and Health Survey data. Sexual and Reproductive Healthcare. 2021;28

8 Islam MZ, Billah A, Islam MM, et al. Negative effects of short birth interval on child mortality in low and middle-income countries: A systematic review and meta-analysis. J Glob Health. 2022; 12:04070

9 Khan KS. Maternal HIV infection increases the risk of adverse perinatal outcomes, especially infant death. Evidence-based Obstetrics & Gynecology. 1999;3(1):97

10 Kihal-Talantikite W, Marchetta GP, Deguen S. Infant mortality related to NO2 and PM exposure: Systematic review and meta-analysis. Int. J. Environ Res Public Health. 2020; 17(8):2623

11 Kim D, Saada A. The social determinants of infant mortality and birth outcomes in Western developed nations: a cross-country systematic review. Int J Environ Res Public Health 2013; 10(6), 2296-2335

12 Lacasana M, Esplugues A, Ballester F. Exposure to ambient air pollution and prenatal and early childhood health effects. European Journal of Epidemiology 2005;20(2):183-99.

13 Lamberti LM, Zakarija-Grković I, Fischer Walker CL, Theodoratou E, Nair H, Campbell H, et al. Breastfeeding for reducing the risk of pneumonia morbidity and mortality in children under two: a systematic literature review and meta-analysis. (Special Issue: The Lives Saved Tool in 2013: New capabilities and applications.). BMC Public Health. 2013;13:S18

14 McDonald CM, Olofin I, Flaxman S, Fawzi WW, Spiegelman D, Caulfield LE, et al. The effect of multiple anthropometric deficits on child mortality: meta-analysis of individual data in 10 prospective studies from developing countries. Am J Clin Nutr. 2013 Apr;97(4):896-901.

15 Musyoka A. Factors associated with under-five child mortality in Kenya: a systematic review and meta-analysis [thesis]. University of Nairobi; 2021

16 O'Hare B, Makuta I, Chiwaula L, Bar-Zeel N. Income and child mortality in developing countries: a systematic review and meta-analysis. J R Soc Med. 2013;106(10):408-14.

17 Ortigoza AF, Granados JAT, Miranda JJ, Alazraqui M, Higuera D, Villamonte G. Characterising variability and predictors of infant mortality in urban settings: findings from 286 Latin American cities. J Epidemiol Community Health. 2021;75(3):264-70.

18 Pillai VK, Bandyopadhyay S. Age effects on infant mortality controlling for race: a meta-analytical study. Health Care Women Int. 1997;18:115-26.

19 Purssell E, Collin J. Fever phobia: The impact of time and mortality – A systematic review and meta-analysis. Int J Nurs Stud. 2016;17:81-9.

20 Rowe RE, Garcia J, Macfarlane AJ, et al. Does poor communication contribute to stillbirths and infant deaths? A review. doi: 10.1093/pubmed/23.1.23

21 Rutherford ME, Mulholland K, Hill PC. How access to health care relates to under-five mortality in sub-Saharan Africa: systematic review. Trop Med Int Health. 2010;15(5):508-19.

22 Sankar MJ, Bireshwar S, Ranadip C, Bhandari N, Taneja S, Martines J. Optimal breastfeeding practices and infant and child mortality: a systematic review and meta-analysis. Acta Paediatrica. 2015;104:3-13.

23 Shi T, Vennard S, Mahdy S, et al. Risk Factors for Poor Outcome or Death in Young Children With Respiratory Syncytial Virus-Associated Acute Lower Respiratory Tract Infection: A Systematic Review and Meta-Analysis. Journal of Infectious Diseases 2022;226:S10-S16.

24 Effect of breastfeeding on infant and child mortality due to infectious diseases in less developed countries: a pooled analysis. Lancet. 2000;355(9202):451-55.

25 Wendt A, Gibbs CM, Peters S, Hogue CJ. Impact of increasing inter-pregnancy interval on maternal and infant health. Paediatr Perinat Epidemiol. 2012;26(suppl 1):239-258.

26 Wigle DT, Arbuckle TE, Turner MC, Bérubé A, Yang Q, Liu S, Krewski D. Epidemiologic evidence of relationships between reproductive and child health outcomes and environmental chemical contaminants. J Toxicol Environ Health B Crit Rev. 2008;11(5-6):373-517.

27 Xu Z, Sheffield PE, Su H, Wang X, Bi Y, Tong S. The impact of heat waves on children's health: a systematic review. Int J Biometeorol. 2014;58:239-47.

28 Balaj M, York HW, Sripada K, Besnier E, Vonen HD, Aravkin A, et al: Parental education and inequalities in child mortality: a global systematic review and meta-analysis. Lancet. 2021;398(10300):608-620.

29 Bhusal MK, Khanal SP. A Systematic Review of Factors Associated with Under-Five Child Mortality. Biomed Res Int. 2022;2022:1181409.

30 Chikhungu LC, Newell ML, Rollins N. Under-five mortality according to maternal survival: a systematic review and meta-analysis. Bull World Health Organ. 2017;95(4):281-287.

31 Forde I, Tripathi V: Association of place of residence and under-five mortality in middle-and low-income countries: A meta-analysis. Children. 2018;5(4):51

32 Garoma S, Fantahun M, Worku A. The effect of intimate partner violence against women on under- five children mortality: A systematic review and meta-analysis. Ethiop Med J. 2011;49(4):331-339.

33 Huang Y, Shallcross D, Pi L, Tian F, Pan J, Ronsmans C. Ethnicity and maternal and child health outcomes and service coverage in western China: a systematic review and meta-analysis. Lancet Glob Health. 2018; 6: e39-e56.
34 Karimi B, Shokrinezhad B. Air pollution and mortality among infant and children under five years: a systematic review and meta-analysis. Atmos Pollut Res. 2020;11(6):61-70.
35 Pretorius CE, Asare H, Genuneit J, Kruger HS, Ricci C: Impact of breastfeeding on mortality in sub-Saharan Africa: a systematic review, meta-analysis, and cost-evaluation. Eur J Pediatr 2020; 179(8):1213–25.
36 Karami B, Abbasi M, Tajvar M. Determinants of neonatal, infant and child mortalities in Iran: a systematic review. Iran J Public Health. 2024; 53(1):104-115.
37 Adane AA, Bailey HD, Morgan VA, Galbally M, Farrant BM, Marriott R, et al. The impact of maternal prenatal mental health disorders on stillbirth and infant mortality: a systematic review and meta-analysis. Arch Women Ment Health. 2021;24:543-555.
38 Ahrens KA, Nelson H, Stidd RL, Moskosky S, Hutcheon JA. Short interpregnancy intervals and adverse perinatal outcomes in high-resource settings: An updated systematic review. Paediatr Perinat Epidemiol. 2019;33(1):O25-O47.
39 Aune D, Saugstad OD, Henriksen T, Tonstad S. Maternal body mass index and the risk of fetal death, stillbirth, and infant death: A systematic review and meta-analysis. JAMA. 2014;311:1536-1546. 42. Bagade T, Chojenta C, Harris ML, et al. Does gender equality and availability of contraception influence maternal and child mortality? A systematic review. BMJ Sexual & Reproductive Health 2020;46(4):244-53.
40 Bagade T, Chojenta C, Harris ML, Nepal S, Loxton D. Does gender equality and availability of contraception influence maternal and child mortality? A systematic review. BMJ Sex Reprod Healt. 2020;46(4):244-253.
41 Brennan AT, Bonawitz R, Gill CJ, Thea DM, Kleinman M, Useem J, et al. A meta-analysis assessing all-cause mortality in HIV-exposed uninfected compared with HIV-unexposed uninfected infants and children. AIDS. 2016; 30(15):2351-2360.
42 Brocklehurst P, French R. The association between maternal HIV infection and perinatal outcome: A systematic review of the literature and meta-analysis. Br J Obstet Gynaecol. 1998;105:836-48.
43 Dadi AF. A systematic review and meta-analysis of the effect of short birth interval on infant mortality in Ethiopia. PLoS One. 2015;10(5):e0126759.
44 Eltayib RAA, Al-Alawi KS, Wirayuda AAB, Al-Azri M, Chan MF. The impact of sociodemographic, macroeconomic, and health status and resources determinants on infant mortality rates in the Gulf Cooperation Council (GCC) countries: A systematic review and meta-analysis. Journal of Neonatal Nursing. 2023;29(4):620-638.
45 Glinianaia SV, Rankin J, Bell R, Pless-Mulloli T, Howel D. Does particulate air pollution contribute to infant death? A systematic review. Environ Health Perspect. 2004; 112(14):1365-1371.

46 Huo N, Zhang K, Wang L, Wang L, Lv W, Cheng W, Jia G. Association of Maternal Body Mass Index With Risk of Infant Mortality: A Dose-Response Meta-Analysis. Front Pediatr. 2021; 9:650413.

47 Jacques N, de Mola CL, Joseph G, Mesenburg MA, da Silveira MF. Prenatal and postnatal maternal depression and infant hospitalization and mortality in the first year of life: A systematic review and meta-analysis. J Affect Disord. 2019;243:201-208.

48 Jahan S. Poverty and infant mortality in the Eastern Mediterranean region: a meta-analysis. J Epidemiol Community Health. 2008; 62(8):745-751.

49 Kiross GT, Chojenta C, Barker D, Tiruye TY, Loxton D. The effect of maternal education on infant mortality in Ethiopia: A systematic review and meta-analysis. PLoS One. 2019;14(7):e0220076.

50 Kozuki N, Lee AC, Silveira MF, Victora CG, Adair L, Humphrey J. The associations of birth intervals with small-for-gestational-age, preterm, and neonatal and infant mortality: a meta-analysis. BMC Public Health. 2013; 13(Suppl. 3): S3.

51 Luben TJ, Wilkie AA, Krajewski AK, Njie F, Park K, Zelasky S, et al. Short-term exposure to air pollution and infant mortality: A systematic review and meta-analysis. Sci Total Environ. 2023; 898:165522.

52 Mazzone PP, Hogg KM, Weir CJ, Stephen J, Bhattacharya S, Chin RFM. Comparison of Perinatal Outcomes for Women with and Without Epilepsy: A Systematic Review and Meta-analysis. JAMA Neurol. 2023;80(5):484-494.

53 Meehan S, Beck CR, Mair-Jenkins J, Leonardi-Bee J, Puleston R. Maternal obesity and infant mortality: A meta-analysis. Pediatrics. 2014;133(5):863-871.

54 Nguyen DTN, Hughes S, Egger S, LaMontagne DS, Simms K, Castle PE,et al. Risk of childhood mortality associated with death of a mother in low-and-middle-income countries: a systematic review and meta-analysis. BMC public health. 2019;19:1281.

55 Quansah R, Armah FA, Essumang DK, Luginaah I, Clarke E, Marfoh K, et al. Association of arsenic with adverse pregnancy outcomes/infant mortality: a systematic review and meta-analysis. Environ Health Perspect. 2015;123(5):412-421.

56 Tousifur R, Barman MP, Hazarika PJ, Bora K. Association of place of residence and infant mortality in India: a meta-analysis. Int J Agricult Stat Sci. 2021;17(Suppl 1):907-13.

57 Sankar MJ, Sinha B, Chowdhury R, Bhandari N, Taneja S, Martines J, et al. Optimal breastfeeding practices and infant and child mortality: a systematic review and meta-analysis. Acta Paediatr. 2015;104: 3-13.

58 Weightman AL, Morgan HE, Shepherd MA, Kitcher H, Roberts C, Dunstan FD. Social inequality and infant health in the UK: Systematic review and meta-analyses. BMJ Open. 2012;2:e000964.

59 Pieper D, Antoine S-L, Mathes T, Neugebauer EA, Eikermann M: Systematic review finds overlapping reviews were not mentioned in every other overview. J Clin Epidemiol. 2014; 67(4):368-375.
